# Case Report: non-monotonic longitudinal RSWA with tonic–phasic dissociation across serial attended video-polysomnography in isolated REM sleep behaviour disorder

**DOI:** 10.3389/frsle.2026.1816079

**Published:** 2026-08-04

**Authors:** Valentina Gnoni, Nazanin Biabani, Istvan Papp, Camilla Speicher, Adam Birdseye, Sakina Dastagir, Martina Mulas, Monica Puligheddu, Clive Ballard, Luigi Ferini-Strambi, David O'Regan, Ivana Rosenzweig

**Affiliations:** 1 Sleep Disorders Centre, Guy's and St Thomas' NHS Foundation Trust, London, United Kingdom; 2 Sleep and Brain Plasticity Centre, Department of Neuroimaging, Institute of Psychiatry, Psychology and Neuroscience (IoPPN), King's College London, London, United Kingdom; 3 Sleep Disorder Research Center and Neurology Unit, Department of Medical Sciences and Public Health, University of Cagliari, Monserrato, Cagliari, Italy; 4 Clinical and Biomedical Sciences, Faculty of Health and Life Sciences, University of Exeter, Exeter, United Kingdom; 5 IRCCS San Raffaele Scientific Institute and University Vita-Salute San Raffaele, Milan, Italy; 6 Faculty of Life Science and Medicine, School of Basic and Medical Biosciences, King's College London, London, United Kingdom

**Keywords:** case report, dream enactment behavior, isolated REM sleep behavior disorder, phasic RSWA, REM atonia index, REM sleep without atonia, tonic RSWA

## Abstract

**Background:**

Quantitative REM sleep without atonia (RSWA) metrics are increasingly used to characterize isolated REM sleep behavior disorder (iRBD) and as candidate biomarkers. Cohort follow-up often reports rising RSWA, but within-individual trajectories, particularly when tonic and phasic components diverge, remain sparsely documented.

**Methods:**

A man in his mid-seventies with iRBD underwent four attended video-polysomnograms over −6.5 years. Submental EMG was quantified using a REM sleep atonia index (RAI) with pre-specified exclusion of REM mini-epochs contaminated by artifact or physiological activation (arousal-linked activation and respiratory-event adjacency). Tonic and phasic submental RSWA components were extracted concurrently. Diary-anchored interviews around each visit captured enactment frequency, ordinal ratings of dream vividness/recall and sleep-related accidental harm.

**Results:**

Corrected RAI varied non-monotonically (0.268, 0.401, 0.313, 0.463). Tonic and phasic RSWA dissociated: tonic activity declined overall, whereas phasic activity rose early and remained elevated. PSG-night enactment frequency tracked REM sleep opportunity (Spearman ρ = 0.89; *n* = 4) more closely than corrected RAI or tonic/phasic indices. Sleep-related harm was absent at the final visit despite persistent phasic activity.

**Conclusion:**

In this case, longitudinal RSWA did not behave as a single monotone scalar. Component-wise reporting alongside explicit REM sleep opportunity and exclusion accounting may be essential for interpreting apparent longitudinal change and relating electrophysiology to behavioral expression.

## 1 Introduction

Isolated REM sleep behavior disorder is defined by recurrent dream enactment behavior with polysomnographic evidence of REM sleep without atonia (RSWA), in the absence of an established neurological syndrome or an alternative explanation (Iranzo et al., 2016). It is now well established that iRBD confers a high long-term risk of phenoconversion to an α-synucleinopathy, and it is increasingly treated as a window onto prodromal disease biology (Iranzo et al., 2016; Roguski et al., 2020; Iranzo et al., 2014). These observations have driven a rapid expansion of quantitative RSWA methods, both to improve diagnostic reliability and to capture features that may bear prognostic information (Puligheddu et al., 2023).

Yet RSWA is not a unitary phenomenon. The normal paralysis of REM sleep reflects a distributed circuitry in the pontomedullary brainstem and its spinal efferents, modulated by forebrain state control and influenced by arousal systems (Arrigoni et al., 2016). In clinical PSG, this physiology is sampled through a small number of surface electromyography (EMG) channels. The resulting signal comprises at least two separable expressions, sustained (“tonic”) elevation of EMG amplitude and brief (“phasic”) bursts (Frauscher et al., 2012). These components do not necessarily covary. Multi-muscle montages, particularly the Sleep-Innsbruck-Barcelona (SINBAR) configuration, demonstrate that limb and cranial muscles contribute non-redundant information and permit empirically derived normative thresholds (Frauscher et al., 2012). Conversely, chin-anchored measures, often the only signal consistently available longitudinally, provide a pragmatic but incomplete view of RSWA phenotype (Frauscher et al., 2012).

A parallel development has been the automation of RSWA quantification. The REM sleep atonia index and related derivatives compute an atonia proportion from short REM mini-epochs, with intermediate classifications and with signal preprocessing intended to reduce noise (Ferri et al., 2010). Such indices can show limited night-to-night variability, whereas EMG activation counts may fluctuate more widely (Ferri et al., 2013). These properties have helped position automated indices as candidate biomarkers for longitudinal studies. Indeed, cohort follow-up in iRBD has shown group-level increases in tonic and phasic EMG activity over time, interpreted as progressive impairment of REM sleep-atonia circuitry (Iranzo et al., 2009). More recently, tonic RSWA has emerged as a particularly strong predictor of phenoconversion risk (Singh et al., 2023).

Cohort trajectories, however, do not compel monotonic within-individual trajectories. Three considerations are germane. First, longitudinal PSG rarely achieves identical montage and signal quality across years (Ferri et al., 2013, 2010; Frauscher et al., 2012; Iranzo et al., 2011). Secondly, RSWA quantification is susceptible to physiologic contamination and methods vary widely (Puligheddu et al., 2023). Thirdly, clinical expression, the observed dream enactment behavior, depends on factors that extend beyond brainstem disinhibition: dream content, environmental triggers, medication, bed partner surveillance, sleep-related accidental harm-avoidance measures, and the opportunity for REM sleep (McCarter et al., 2015; Dodet et al., 2024; Högl et al., 2018). It follows that the mapping from RSWA to behavior may be gated by ‘REM sleep opportunity' and state stability, rather than by RSWA magnitude alone.

Against this background, we report a single patient with iRBD who underwent four attended video-polysomnograms over approximately six and a half years, accompanied by diary-derived symptom tracking at four peri-PSG windows.

## 2 Methods

This longitudinal case report draws on four attended overnight video-polysomnography studies (V1–V4) performed in routine clinical care, with diary-anchored structured interviews around each study; written informed consent for publication was obtained and dates were coarsened for pseudo-anonymisation (see Supplementary Methods and Supplementary Table 1). Symptom tracking used ordinal diary ratings (enactment frequency, dream vividness/recall, sleep-related harm severity) in pre-specified time windows for within-person description, not as validated severity scales (Supplementary Methods). All vPSGs included standard EEG/EOG/ECG, submental EMG, and respiratory channels scored to contemporaneous AASM criteria (Berry et al., 2012); limb EMG was not comparable across visits and longitudinal inference was anchored to submental EMG. RSWA was quantified using three complementary submental metrics: REM atonia index (RAI) plus tonic and phasic components computed from 3-s mini-epochs/30-s epochs, with a corrected RAI derived after pre-specified exclusion of artifact and physiologic contamination (arousals and respiratory-event adjacency). REM opportunity (analyzed REM minutes, excluded REM sleep %, REM bouts) is reported for each visit (Supplementary Table 1). Exploratory within-person associations used descriptive Spearman correlations (*n* = 4; Supplementary Table 2).

## 3 Case report and results

A man in his mid-seventies with a several-year history of vivid dreaming and recurrent nocturnal motor behaviors was evaluated for suspected REM sleep behavior disorder (Supplementary Table 1). Differential diagnoses considered included REM-predominant OSA with arousal-linked behaviors (pseudo-RBD), NREM parasomnia overlap, nocturnal seizures, and medication-associated sleep behaviors. iRBD was concluded because repeated attended vPSG demonstrated RSWA with clinical histories of dream-concordant enactment, without exposure to agents classically associated with RSWA/complex sleep behaviors, and without features suggestive of epilepsy or primary NREM parasomnia. Episodes reported by both the patient and bed-partner characterized by punching, kicking, thrashing, and vocalization that appeared concordant with dream activity. These episodes had resulted in sleep-related inadvertent harm sustained by both the patient and bed partner. Around each polysomnogram, a structured diary-based interview quantified enactment frequency across four temporal windows (3 months pre-study, 1 month pre-study, the study night, and 1 month post-study) and rated dream vividness, dream recall, and sleep-related harm severity. These domains were summarized using a composite enactment score (CES4D; Supplementary Table 1). Diary data were incomplete for the first study because the diary instrument was implemented after the initial polysomnogram; only the PSG-night rating was available at that time (Supplementary Table 1). Because V3 occurred without PAP and showed pronounced arousal-driven REM fragmentation with a higher proportion of excluded REM sleep, the mid-series dip in corrected RAI should be interpreted as context-dependent rather than as a stable biological reversal. Notably, when V3 is treated as a physiologically confounded visit, corrected RAI increases monotonically from V1 to V2 to V4 (0.268 → 0.401 → 0.463), while tonic RSWA falls and phasic RSWA remains elevated.

Past medical history relevant to the present analysis included obstructive sleep apnoea treated with positive airway pressure (PAP). Melatonin exposure varied across follow-up, reflecting intermittent self-directed cessation and re-initiation; recorded doses at the time of V2–V4 were 2 mg, 6 mg, and 2 mg, respectively (Supplementary Table 1). He was not taking clonazepam, antidepressants (SSRIs/SNRIs/TCAs), antipsychotics, or dopaminergic agents during the peri-PSG windows. His neurologic examination was normal and he did not develop overt parkinsonism during the investigative period. The patient reported that dream enactment behaviors were most disruptive during periods of fragmented sleep, and that perceived night-to-night variability made it difficult to judge whether symptoms were improving. They valued objective PSG feedback but found that a single study did not always reflect their typical weeks.

Four attended vPSGs (V1–V4) were available over approximately 6.5 years. Standard sleep staging and respiratory scoring followed American Academy of Sleep Medicine criteria (Berry et al., 2012), and macrostructure/respiratory indices are summarized in Supplementary Table 1. Sleep efficiency varied across visits, with the lowest efficiency at V2 (60.1%) and otherwise broadly comparable values at V1, V3, and V4 (76.0%, 76.5%, 75.9%). REM proportion was lowest at V2 (14.9% of total sleep time) and higher at V1, V3, and V4 (23.3%, 16.6%, and 25.0%, respectively) (Supplementary Table 1). Arousal burden was notably increased at V3 (arousal index 36.2/h) relative to the other visits (15.0/h, 9.5/h, 12.7 /h) (Supplementary Table 1). The V3 recording was inadvertently performed without PAP owing to a misunderstanding, coinciding with a recorded apnoea–hypopnoea index (20.5/h) and marked REM sleep instability, reflected by an increased number of REM bouts (*n* = 46) (Supplementary Table 1). This visit therefore represents a clinically interpretable but confounded physiological context in which arousal-driven REM sleep fragmentation was prominent.

**Figure 1:**
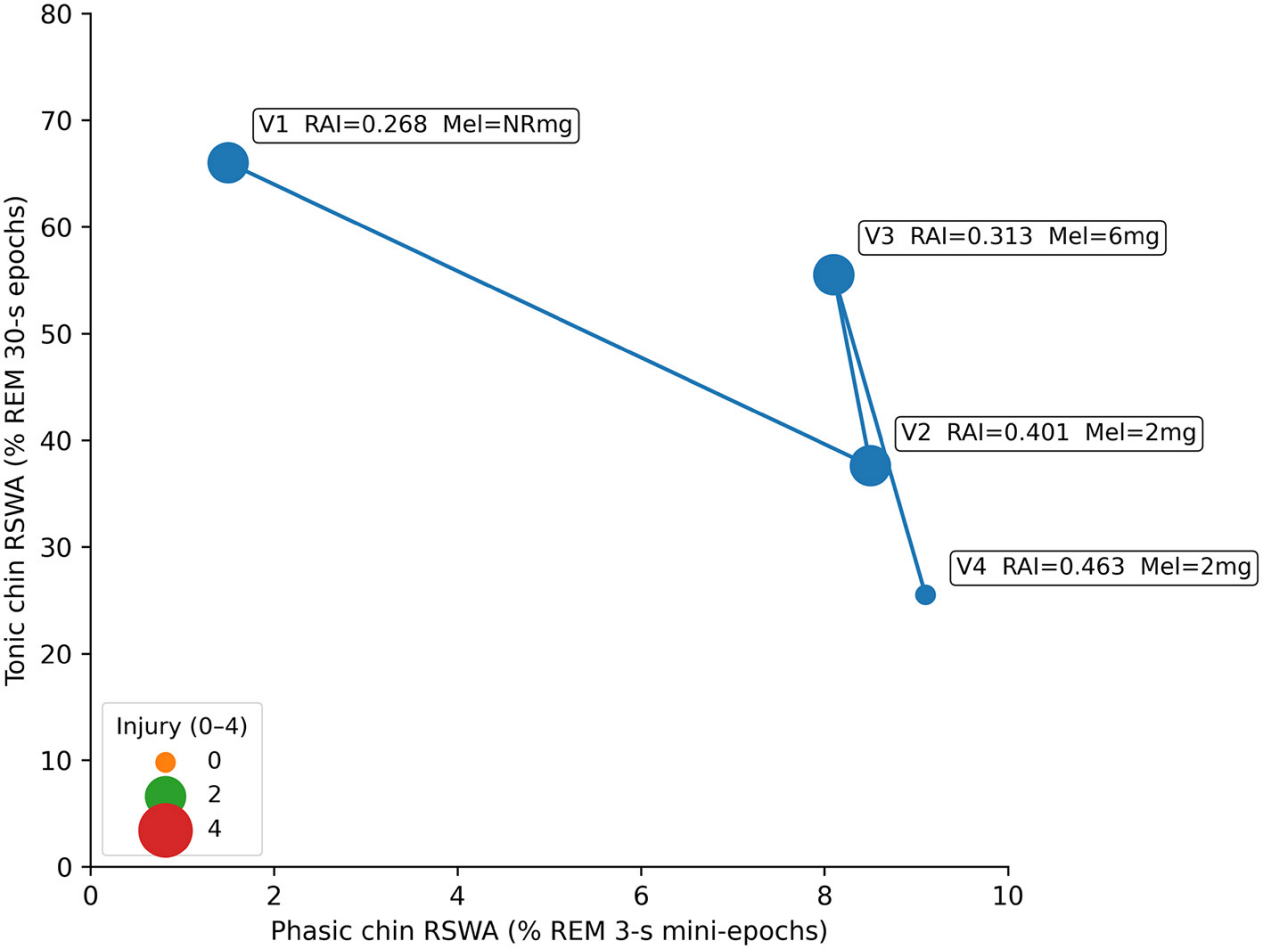
Longitudinal shift in submental RSWA phenotype in tonic–phasic space, with concurrent sleep-related inadvertent harm severity. Each point represents one attended overnight video-polysomnogram (vPSG) (V1–V4) plotted in a phase plane defined by phasic (x-axis) and tonic (y-axis) submental REM sleep without atonia (RSWA). Phasic submental RSWA is expressed as the percentage of analyzed REM sleep 3-s mini-epochs containing ≥1 phasic EMG burst. Tonic submental RSWA is expressed as the percentage of analyzed REM sleep 30-s epochs meeting tonic criteria. Points are connected in chronological order to indicate the direction of follow-up (time spacing not to scale). Marker area is proportional to sleep-related harm severity (ordinal diary-derived score, 0–4) anchored to each vPSG (inset size key). Each callout lists the visit identifier, corrected REM atonia index (RAI; 0–1), and melatonin dose (mg) at the time of the study, facilitating cross-reference to Table 2 and Supplementary Table 1. Quantification was performed using an identical longitudinal submental EMG extraction pipeline across visits; REM segments excluded because of non-physiological artifact or physiological contamination (including arousal-linked activation and respiratory-event adjacency) were removed prior to computation (see Table 2 and Supplementary Table 1). EMG, electromyography; RAI, REM atonia index; REM, rapid eye movement; RSWA, REM sleep without atonia; vPSG, video-polysomnography.

**Figure 2:**
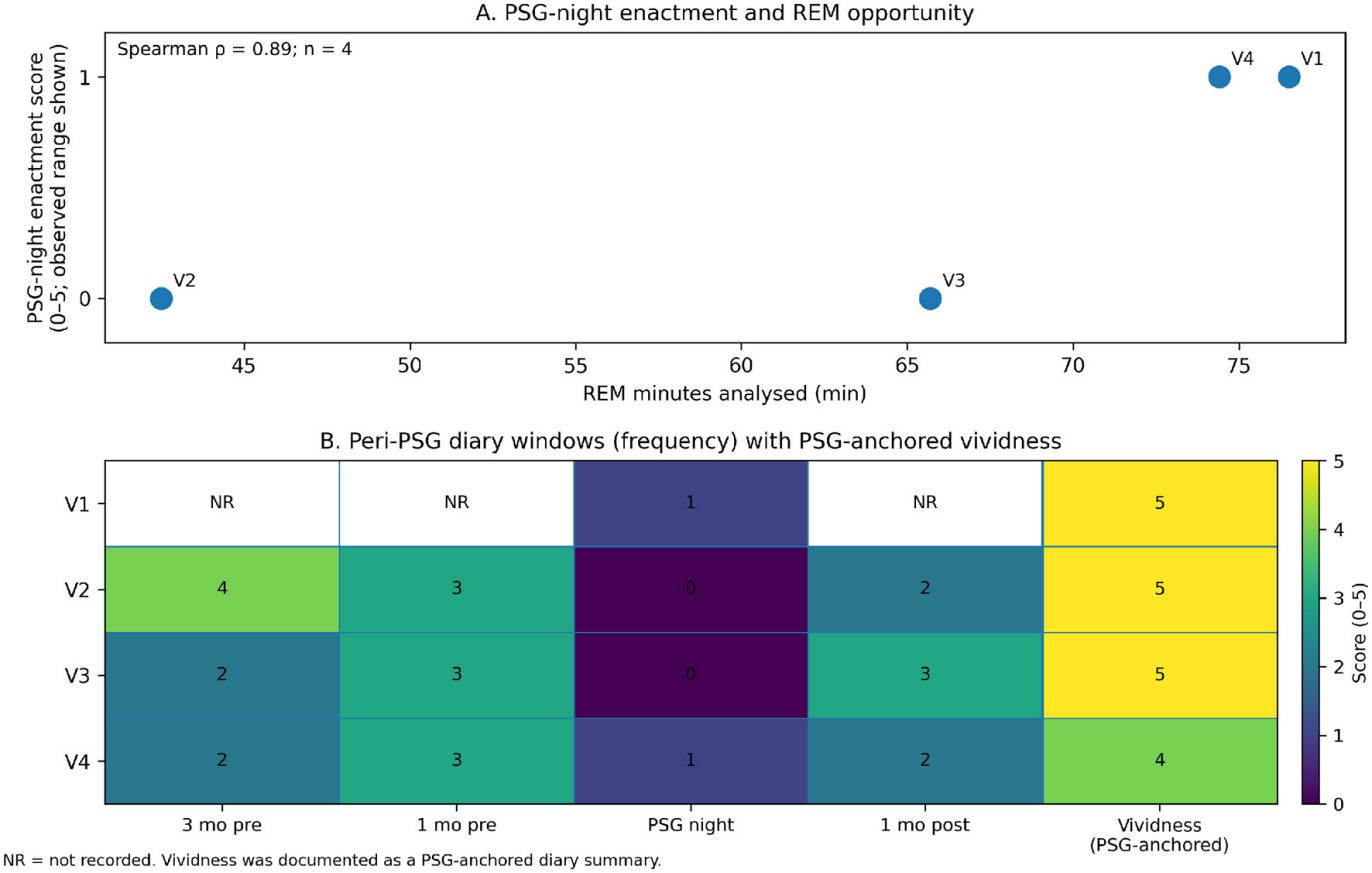
REM opportunity and peri-PSG symptom sampling. **(A)** PSG-night dream enactment frequency (F_PSG, ordinal 0–5 scale) plotted against REM minutes analyzed for RSWA/RAI quantification across four attended video-polysomnograms (V1–V4). Each point represents one vPSG and is labeled by visit. The Spearman rank correlation coefficient (ρ) and *n* are shown; no *p*-value is presented. For legibility, the y-axis is restricted to the observed PSG-night scores in this case (0–1); the underlying diary scale is 0–5. **(B)** Diary matrix summarizing peri-PSG behavioral sampling. Rows correspond to visits (V1–V4). Columns report diary-derived enactment frequency (0–5) for four windows anchored to each vPSG (3 months pre-vPSG, 1 month pre-vPSG, vPSG night, and 1 month post-vPSG), plus a final column showing dream vividness (0–5) recorded as a PSG-anchored diary summary (not time-resolved across windows). Cell shading encodes the raw ordinal score (0–5), with the numeric value printed within each cell; NR denotes not recorded (diary not instituted until after V1 for the pre/post windows). The vertical divider separates the time-resolved peri-PSG windows from the PSG-anchored vividness measure. F_PSG, PSG-night enactment frequency score; NR, not recorded; PSG, polysomnogram; REM, rapid eye movement; RSWA, REM sleep without atonia; vPSG, video-polysomnography.

Across visits, quantitative REM atonia and RSWA components showed non-monotonic trajectories. Corrected RAI values were 0.268 (V1), 0.401 (V2), 0.313 (V3), and 0.463 (V4) (Supplementary Table 1). The tonic and phasic components were partially dissociated: tonic chin activity declined overall across the series (66.0%, 37.6%, 55.7%, 25.7%), whereas phasic chin activity rose from baseline and remained elevated (1.5%, 8.5%, 8.1%, 9.1%) (Supplementary Table 1; Figure 1). The proportion of REM sleep excluded from quantitative analysis due to artifact/contamination was minimal at V1–V2 (0%) and V4 (0.2%), but higher at V3 (14.3%), consistent with the more fragmented and arousal-rich recording (Supplementary Table 1). REM sleep minutes contributing to quantitative analysis were 76.5, 42.5, 65.7, and 74.4 min at V1–V4, respectively (Supplementary Table 1), providing context for interpretation of longitudinal RSWA estimates and for the “REM sleep opportunity” concept illustrated in Figure 2.

Clinically, diary-derived enactment burden was broadly stable from V2 to V4 (total enactment burden 2.4, 2.0, and 2.1 on a 0–5 scale), while harm severity remained 2/4 at V1–V3 but fell to 0/4 at V4 (Supplementary Table 1). These diary-derived ratings are presented as within-person descriptive measures rather than as validated severity outcomes. Notably, no sleep-related harm was reported at V4, which coincided with the highest corrected RAI and persistent high phasic RSWA, emphasizing that behavioral harm and quantitative RSWA are related but not interchangeable constructs in longitudinal assessment (Figures 1, 2; Supplementary Table 1).

## 4 Discussion

RSWA is the electrophysiological hallmark required for the diagnosis of RBD, yet it is not a single physiological quantity. On contemporary definitions, RSWA is expressed as sustained (tonic) elevation of EMG activity during REM sleep and/or excessive transient (phasic) bursts, typically quantified in submental EMG with optional limb channels (Puligheddu et al., 2023) (Table 1). The practical problem for biomarker work is that tonic and phasic RSWA may index partly separable elements of REM motor dyscontrol, and the linkage between RSWA magnitude and overt dream enactment behavior (DEB) is neither direct nor stable across disorders, stages, montages, or nights.

**Table 1:** Selected longitudinal and methodological literature relevant to RSWA quantification in iRBD.

Citation	Design/sample	Muscles/montage	RSWA metric(s)	Key message
Iranzo et al. (2009)	Idiopathic RBD; longitudinal PSG follow-up	Study-specific chin/limb EMG	Tonic/phasic EMG activity	Group-level RSWA increases reported; does not entail monotonic within-individual trajectories.
Ferri et al. (2010)	Methodological development	Submental EMG	Atonia index/RAI framework	Automated atonia metrics depend on preprocessing and signal quality; artifact handling is central.
Ferri et al. (2013)	Night-to-night variability	Submental EMG	Atonia index and activation measures	Atonia index shows modest variability; activation measures vary more, supporting component-wise reporting.
Frauscher et al. (2012)	Normative EMG values; SINBAR montage	Multi-muscle montage (SINBAR)	Tonic and phasic thresholds	Montage selection materially influences RSWA detection; limb muscles add information beyond chin alone.

iRBD, isolated REM sleep behavior disorder; PSG, polysomnogram; RAI, REM atonia index; REM, rapid eye movement; RSWA, REM sleep without atonia; SINBAR, Sleep Innsbruck Barcelona montage.

### 4.1 RSWA morphology and dream enactment: what is established

At the level of event generation, the best evidence indicates that complex motor–behavioral episodes in RBD occur disproportionately during phasic REM sleep periods, rather than during tonic REM sleep. In a structured vPSG analysis, the more complex episodes were enriched for rapid eye movements and transient EEG phenomena (including alpha bursts and sawtooth waves), consistent with a state of increased REM sleep microstructural activation immediately preceding enactment (Manni et al., 2009). This view is convergent with work emphasizing the relation between abnormal behaviors and REM sleep microstructure, in which behaviors cluster with phasic REM sleep features (Frauscher et al., 2009). More recent EEG work in iRBD has identified reproducible cortical dynamics in the period preceding DEBs, supporting the notion that cortical state and REM sleep microstructure contribute materially to whether motor disinhibition becomes overt behavior (Date et al., 2024).

At the level of signal detection, the SINBAR programme is relevant because it ties phasic muscle activity to the practical capture of clinical manifestations. The SINBAR montage (mentalis, flexor digitorum superficialis, extensor digitorum brevis) was developed empirically to provide normative EMG values and optimize diagnostic discrimination (Frauscher et al., 2012). In subsequent work, simultaneous recording of these muscles detected the large majority of motor and vocal manifestations, and increased phasic EMG was proposed as an efficient trigger for targeted video review (Iranzo et al., 2011). These observations are often read as indirect support for the primacy of phasic activity in behavioral expression.

Two limits should be stated. First, these studies speak principally to the timing of episodes and the efficiency of detection, not to the biological meaning of RSWA as a marker of neurodegenerative burden. Secondly, they do not establish a monotone relationship between phasic RSWA magnitude and the severity or injuriousness of DEB; the episode-level literature supports phasic REM as the favored context for enactment. However, the clinical severity of enactment likely reflects additional factors, many of which are not measured by chin EMG metrics alone.

### 4.2 RSWA as a biomarker of underlying pathology

When RSWA is used as a biomarker, either for phenoconversion risk in iRBD or for staging within established synucleinopathy, the literature is more heterogenous. In a large iRBD cohort study, tonic REM sleep muscle activity at baseline was the strongest predictor of phenoconversion risk (Singh et al., 2023). Independent longitudinal work has likewise supported RSWA severity as a risk-stratification marker, though with methodological heterogeneity in the definition of RSWA (McCarter et al., 2019). A contemporary review of predictors of progression notes that tonic and phasic RSWA may reflect different pathophysiological alterations within brainstem REM circuitry and summarizes the emerging but not yet settled evidence on their differential predictive roles (de Natale et al., 2022).

A separate, clinically important observation is that RSWA and subjective DEB severity can decouple in established Parkinson disease. In a longitudinal vPSG study, approximately one quarter of patients reported subjective improvement of RBD symptoms, whilst all RSWA measures increased significantly at follow-up (Figorilli et al., 2020). This result does not prove that DEB disappears whilst RSWA progresses in every patient. However, it does provide a concrete precedent for the scenario that clinicians recognize, clinical expression may attenuate without parallel reduction in RSWA.

The interpretive consequence is that the field should be cautious about treating any single RSWA summary measure as a complete representation of the state of REM sleep motor control. This is also relevant for atonia indices such as the REM Atonia Index/RAI, which were designed to quantify the proportion of REM time spent in low-amplitude submental EMG and have strong validation for diagnostic use (Figorilli et al., 2017; Ferri et al., 2010, 2008). In principle, an atonia index can improve if sustained background tone diminishes, even if short phasic bursts persist or increase, because those bursts occupy a small fraction of REM time. In this case corrected RAI improved at the final timepoint whilst phasic chin activity remained high (Figure 1; Table 2), a pattern that is physiologically plausible within the framework and clinically consequential for how longitudinal improvement is interpreted.

**Table 2:** RSWA pipeline transparency across visits (submental-anchored analysis).

Parameter	V1	V2	V3	V4
REM sleep minutes analyzed (min)	76.5	42.5	65.7	74.4
REM sleep excluded due to artifact/contamination (%)	0	0	14.3	0.2
Number of REM bouts	7	9	46	31
REM atonia index, corrected (RAI)	0.268	0.401	0.313	0.463

“REM sleep excluded” reflects REM sleep mini-epochs removed by pre-specified exclusion rules for artifact and physiologic contamination. REM sleep bouts count provides a descriptive index of REM sleep fragmentation across the night (bout = contiguous REM sleep separated by non-REM or wake).

Two brief clinical modifiers are also worth noting. Intermittent melatonin exposure in RBD has been suggested to preferentially reduce tonic manifestations of RSWA, and may thereby theoretically exaggerate tonic–phasic dissociation in some patients (Takeuchi et al., 2001; Kunz and Mahlberg, 2010). Similarly, the V3 “dip” in corrected RAI occurred on a night with PAP omission, high arousal burden, and heavy REM sleep fragmentation. Given evidence that the elevated EMG milieu of RBD can interact with OSA severity and REM sleep physiology, respiratory instability should be treated as a first-order confounder when interpreting apparent longitudinal change in RSWA indices (Huang et al., 2011).

### 4.3 iRBD, phenoconverted RBD, and RSWA without RBD: stage and etiology matter

RBD in mid-to-late life, when confirmed on vPSG, is strongly associated with prodromal α-synucleinopathy, and this framing has been formalized in high-level reviews and biomarker-oriented position pieces (Miglis et al., 2021; Högl et al., 2018; Dauvilliers et al., 2018). Yet two complications are now well documented.

First, RSWA can occur without clinical RBD, particularly in early-stage Parkinson disease. A recent Movement Disorders analysis emphasized that isolated RSWA is not equivalent to RBD in early PD, reinforcing that the electrophysiological substrate may be present without manifest enactment behavior (Dodet et al., 2024). This is directly germane to the question of disappearing re-enactments. The absence of overt episodes does not, by itself, adjudicate the status of the REM sleep atonia network.

Secondly, RSWA is not exclusive to synucleinopathy. In probable Alzheimer disease, one polysomnographic series concluded that RBD was rare, but RSWA was relatively frequent (Gagnon et al., 2006). The biological interpretation remains uncertain but it nevertheless cautions against assuming that RSWA morphology always maps to the same upstream lesion across disorders.

Medication-associated RSWA provides a further example of aetiological heterogeneity. Serotonergic antidepressants have been associated with increased submental EMG tone during REM sleep, with some work suggesting a preferential effect on tonic rather than phasic components (Winkelman and James, 2004). In a large clinical cohort, antidepressant treatment was associated with elevated RSWA without necessarily meeting full RBD criteria. Importantly, differences in RSWA distribution/type were observed between antidepressant-treated patients and Parkinson disease-RBD patients, with relatively greater anterior tibialis RSWA in the former and higher tonic RSWA in the latter (McCarter et al., 2015). Thus, RSWA likely reflects multiple mechanisms, and the muscles sampled may determine which is most apparent.

### 4.4 Why the field still lacks a settled RSWA biomarker

The dominance of chin EMG in routine practice is pragmatic, but it is not necessarily optimal for biomarker inference. SINBAR-derived work demonstrates that combining mentalis with limb muscles increases the capture of phasic activity and improves linkage to behavioral manifestations (Frauscher et al., 2012; Iranzo et al., 2011). Software implementations of SINBAR criteria continue to operationalise this multi-muscle approach (Röthenbacher et al., 2022) and recent work suggests that certain phasic limb metrics show excellent discriminative performance (Frauscher et al., 2012; Cesari et al., 2023).

What remains poorly established is whether different muscle groups and RSWA morphologies exhibit different temporal trajectories across the prodrome and after phenoconversion. If tonic and phasic components can diverge within a single muscle (Figure 1), it is also plausible that divergence may be magnified across muscle groups, given that cranial and limb musculature are governed by partially distinct motor pools and may be differentially influenced by brainstem and spinal mechanisms. The field presently has limited longitudinal multi-muscle data with stable montage and transparent artifact handling sufficient to resolve this.

### 4.5 Where this case sits and what it adds

Three observations from this case can be similarly compared against the state of knowledge. Firstly, atonia indices can improve whilst phasic RSWA persists. This is consistent with what an atonia index measures (Ferri et al., 2010, 2008). It follows that improvement in a global atonia metric does not imply resolution of phasic disinhibition, and component-wise reporting is not optional if one wishes to interpret directionality (Figure 1; Table 2). Secondly, PSG-night DEB capture is constrained by REM opportunity. In this patient, PSG-night enactment frequency tracked REM sleep duration far more closely than it tracked tonic/phasic/RAI measures (Supplementary Table 1; Figure 2B). This sits comfortably with the episode-level literature locating DEB in phasic REM sleep microstructure (Manni et al., 2009) and with the broader recognition that vPSG samples a limited and state-dependent portion of a variable phenotype. And finally, this case illustrates the DEB–RSWA dissociation problem. Longitudinal PD data demonstrate that subjective DEB improvement can coexist with increasing RSWA (Figorilli et al., 2020). The present case adds a mechanistically coherent variant: a global atonia metric improved while phasic activity persisted, and behavioral sampling varied across peri-PSG windows.

When dream re-enactment attenuates, whether by progression, adaptation, or state change, does RSWA attenuate, remain stable, or redistribute across tonic/phasic and across muscle groups? The answer remains unknown but a parsimonious explanation for such dissociation could be that the determinants of RSWA and the determinants of overt enactment are not identical. A further possibility, necessarily speculative, is that the dissociation may reflect variation in suprapontine gating rather than variation in the brainstem substrate alone. Motor–behavioral episodes in RBD occur preferentially during phasic REM sleep and frequently coincide with phasic EEG/EOG phenomena, consistent with transient cortical activation immediately upstream of enactment (Manni et al., 2009; Date et al., 2024). In Parkinson disease, subjective attenuation of RBD behaviors has been reported despite increasing RSWA on follow-up, implying that behavioral expression can diminish without recovery of REM atonia (Figorilli et al., 2020). Recent semiological work comparing iRBD with narcolepsy-related RBD, a context of orexin deficiency, demonstrates categorical differences in motor topography and complexity, supporting the broader proposition that neuromodulatory milieu and suprapontine integration may shape which motor patterns are released when REM atonia fails (Drakatos et al., 2025). Under such a scheme, longitudinal change in RSWA may not be mirrored by longitudinal change in DEB, and muscle-specific trajectories may differ by etiology. This position remains hypothetical and its value is that it makes testable predictions about measurement. For example, if enactment is preferentially released during particular REM sleep microstates, then component-wise RSWA should be reported alongside REM sleep microstructure and phasic EEG/EOG context.

Several uncertainties follow from the present state of evidence and known unknowns include the extent to which tonic vs. phasic RSWA differentially predicts phenoconversion across cohorts (Singh et al., 2023; de Natale et al., 2022); whether RSWA trajectories differ before and after phenoconversion; and whether muscle-group involvement shifts systematically with disease stage or etiology (Frauscher et al., 2012; McCarter et al., 2015). A plausible class of unrecognized determinants concerns REM sleep microstructure and cortical state. Episode-level evidence implicates phasic REM sleep features and transient EEG phenomena in the immediate prelude to DEB (Manni et al., 2009; Date et al., 2024). Yet most RSWA biomarker work remains anchored to EMG summaries. It is therefore possible that part of the apparent inconsistency across cohorts reflects a mismatch between what is quantified (global EMG summaries) and what precipitates behavior (a conjunction of disinhibition with REM sleep microstructure and cortical activation).

The case supports a restrained methodological position. Longitudinal RSWA reporting intended to inform biomarker inference should, at minimum, present tonic and phasic measures alongside an atonia index, and should state the montage, the amount of REM sleep analyzed, and the fraction excluded for contamination (Puligheddu et al., 2023). Where feasible, stable multi-muscle recording informed by SINBAR experience should be adopted, since it improves capture of phasic activity linked to behavioral manifestations and may reveal differential muscle trajectories (Frauscher et al., 2012; Iranzo et al., 2011; Röthenbacher et al., 2022). Finally, if the aim is to relate RSWA to behavioral severity, then peri-PSG behavioral sampling and REM sleep microstructure metrics ought to be treated as first-order variables rather than as incidental context.

### 4.6 Take-home message

In conclusion, in this case report, using a transparent longitudinal series, we demonstrate that RSWA components and behavioral expression can separate in time and direction, and that this separation is consistent with established event-level physiology and with longitudinal observations in Parkinson disease. Moreover, we advance that these findings may suggest that RSWA should be treated as a composite phenotype whose meaning is contingent upon component, muscle, state, and stage.

## Data Availability

The original contributions presented in the study are included in the article/Supplementary material, further inquiries can be directed to the corresponding author.
