# Supplementary material for: Case Report: non-monotonic longitudinal RSWA with tonic–phasic dissociation across serial attended video-polysomnography in isolated REM sleep behaviour disorder
Source: Front Sleep. 2026 Aug 4;5:1816079. doi: 10.3389/frsle.2026.1816079 (PMC13481305; doi:10.3389/frsle.2026.1816079)
Supplement: Supplementary file 1 [file Supplementary_file_1.pdf]

Case Report: Non-monotonic longitudinal RSWA with tonic–phasic dissociation across serial attended video-polysomnography in isolated REM sleep behaviour disorder

Running title: Longitudinal RSWA non-monotonicity in iRBD

Valentina Gnoni[1,2], Nazanin Biabani[2], Istvan Papp[2], Camilla Speicher[2], Adam Birdseye[1], Sakina Dastagir[1], Martina Mulas[3], Monica Puligheddu[3], Clive Ballard[4], Luigi Ferini-Strambi5, David O'Regan[1,6], Ivana Rosenzweig[1,2]

Affiliations

1. Sleep Disorders Centre, Guy's and St Thomas' NHS Foundation Trust, London, UK

2. Sleep and Brain Plasticity Centre, Department of Neuroimaging, Institute of Psychiatry, Psychology and Neuroscience (IoPPN), King's College London, London, UK

3. Sleep Disorder Research Center and Neurology Unit, Department of Medical Sciences and Public Health, University of Cagliari, Monserrato, Cagliari, Italy

4. Clinical and Biomedical Sciences, Faculty of Health and Life Sciences, University of Exeter, Exeter, UK

5. IRCCS San Raffaele Scientific Institute, Department of Clinical Neurosciences, Neurology-Sleep Disorders Center, Milan, Italy.

6. Faculty of Life Science and Medicine, School of Basic and Medical Biosciences, King's College London, London, United Kingdom

Corresponding author: Ivana Rosenzweig (1, 2); Email: ivana.1.rosenzweig@kcl.ac.uk

Main manuscript: 2 Figures (Fig 1–2); 2 Tables (Table 1–2)

Supplement: 3 Supplementary Tables (S0–S2)

Abstract

Background: Quantitative REM sleep without atonia (RSWA) metrics are increasingly used to characterise isolated REM sleep behaviour disorder (iRBD) and as candidate biomarkers. Cohort follow-up often reports rising RSWA, but within-individual trajectories, particularly when tonic and phasic components diverge, remain sparsely documented.

Methods: A man in his mid-seventies with iRBD underwent four attended video-polysomnograms over ~6.5 years. Submental EMG was quantified using a REM sleep atonia index (RAI) with pre-specified exclusion of REM mini-epochs contaminated by artefact or physiological activation (arousal-linked activation and respiratory-event adjacency). Tonic and phasic submental RSWA components were extracted concurrently. Diary-anchored interviews around each visit captured enactment frequency,ordinal ratings of dream vividness/recall and sleep-related accidental harm.

Results: Corrected RAI varied non-monotonically (0.268, 0.401, 0.313, 0.463). Tonic and phasic RSWA dissociated: tonic activity declined overall, whereas phasic activity rose early and remained elevated. PSG-night enactment frequency tracked REM sleep opportunity (Spearman $\rho=0.89$; $n=4$) more closely than corrected RAI or tonic/phasic indices. Sleep-related harm was absent at the final visit despite persistent phasic activity.

Conclusion: In this case, longitudinal RSWA did not behave as a single monotone scalar. Component-wise reporting alongside explicit REM sleep opportunity and exclusion accounting may be essential for interpreting apparent longitudinal change and relating electrophysiology to behavioural expression.

Key words: case report; Isolated REM sleep behaviour disorder; REM sleep without atonia; REM atonia index; tonic RSWA; phasic RSWA; dream enactment behaviour.

Introduction

Isolated REM sleep behaviour disorder is defined by recurrent dream enactment behaviour with polysomnographic evidence of REM sleep without atonia (RSWA), in the absence of an established neurological syndrome or an alternative explanation (Iranzo et al., 2016). It is now well established that iRBD confers a high long-term risk of phenoconversion to an α-synucleinopathy, and it is increasingly treated as a window onto prodromal disease biology (Iranzo et al., 2016, Roguski et al., 2020, Iranzo et al., 2014). These observations have driven a rapid expansion of quantitative RSWA methods, both to improve diagnostic reliability and to capture features that may bear prognostic information (Puligheddu et al., 2023).

Yet RSWA is not a unitary phenomenon. The normal paralysis of REM sleep reflects a distributed circuitry in the pontomedullary brainstem and its spinal efferents, modulated by forebrain state control and influenced by arousal systems (Arrigoni et al., 2016). In clinical PSG, this physiology is sampled through a small number of surface electromyography (EMG) channels. The resulting signal comprises at least two separable expressions, sustained ('tonic') elevation of EMG amplitude and brief ('phasic') bursts (Frauscher et al., 2012). These components do not necessarily covary. Multi-muscle montages, particularly the Sleep-Innsbruck-Barcelona (SINBAR) configuration, demonstrate that limb and cranial muscles contribute non-redundant information and permit empirically derived normative thresholds (Frauscher et al., 2012). Conversely, chin-anchored measures, often the only signal consistently available longitudinally, provide a pragmatic but incomplete view of RSWA phenotype (Frauscher et al., 2012).

A parallel development has been the automation of RSWA quantification. The REM sleep atonia index and related derivatives compute an atonia proportion from short REM mini-epochs, with intermediate classifications and with signal preprocessing intended to reduce noise (Ferri et al., 2010). Such indices can show limited night-to-night variability, whereas EMG activation counts may fluctuate more widely (Ferri et al., 2013). These properties have helped position automated indices as candidate biomarkers for longitudinal studies. Indeed, cohort follow-up in iRBD has shown group-level increases in tonic and phasic EMG activity over time, interpreted as progressive impairment of REM sleep-atonia circuitry (Iranzo et al., 2009). More recently, tonic RSWA has emerged as a particularly strong predictor of phenoconversion risk (Singh et al., 2023).

Cohort trajectories, however, do not compel monotonic within-individual trajectories. Three considerations are germane. First, longitudinal PSG rarely achieves identical montage and signal quality across years (Ferri et al., 2013, Ferri et al., 2010, Frauscher et al., 2012, Iranzo et al., 2011). Secondly, RSWA quantification is susceptible to physiologic contamination and methods vary widely(Puligheddu et al., 2023). Thirdly, clinical expression, the observed dream enactment behaviour, depends on factors that extend beyond brainstem disinhibition: dream content, environmental triggers, medication, bed partner surveillance, sleep-related accidental harm-avoidance measures, and the opportunity for REM sleep(McCarter et al., 2015, Dodet et al., 2024, Högl et al., 2018). It follows that the mapping from RSWA to behaviour may be gated by 'REM sleep opportunity' and state stability, rather than by RSWA magnitude alone.

Against this background, we report a single patient with iRBD who underwent four attended video-polysomnograms over approximately six and a half years, accompanied by diary-derived symptom tracking at four peri-PSG windows.

Methods

This longitudinal case report draws on four attended overnight video-polysomnography studies (V1–V4) performed in routine clinical care, with diary-anchored structured interviews around each study; written informed consent for publication was obtained and dates were coarsened for pseudo-anonymisation (see Supplementary Methods and Supplementary Table S1). Symptom tracking used ordinal diary ratings (enactment frequency, dream vividness/recall, sleep-related harm severity) in pre-specified time windows for within-person description, not as validated severity scales (Supplementary Methods). All vPSGs included standard EEG/EOG/ECG, submental EMG, and respiratory channels scored to contemporaneous AASM criteria(Berry et al., 2012); limb EMG was not comparable across visits and longitudinal inference was anchored to submental EMG. RSWA was quantified using three complementary submental metrics: REM atonia index (RAI) plus tonic and phasic components computed from 3-s mini-epochs/30-s epochs, with a corrected RAI derived after pre-specified exclusion of artefact and physiologic contamination (arousals and respiratory-event adjacency). REM opportunity (analysed REM minutes, excluded REM sleep %, REM bouts) is reported for each visit (Supplementary Table S1). Exploratory within-person associations used descriptive Spearman correlations (n=4; Supplementary Table S2).

Case Report and Results

A man in his mid-seventies with a several-year history of vivid dreaming and recurrent nocturnal motor behaviours was evaluated for suspected REM sleep behaviour disorder (Supplementary Table S0). Differential diagnoses considered included REM-predominant OSA with arousal-linked behaviours (pseudo-RBD), NREM parasomnia overlap, nocturnal seizures, and medication-associated sleep behaviours. iRBD was concluded because repeated attended vPSG demonstrated RSWA with clinical histories of dream-concordant enactment, without exposure to agents classically associated with RSWA/complex sleep behaviours, and without features suggestive of epilepsy or primary NREM parasomnia. Episodes reported by both the patient and bed-partner characterized by punching, kicking, thrashing, and vocalisation that appeared concordant with dream activity. These episodes had resulted in sleep-related inadvertent harm sustained by both the patient and bed partner. Around each polysomnogram, a structured diary-based interview quantified enactment frequency across four temporal windows (3 months pre-study, 1 month pre-study, the study night, and 1 month post-study) and rated dream vividness, dream recall, and sleep-related harm severity. These domains were summarised using a composite enactment score (CES4D; Supplementary Table S1). Diary data were incomplete for the first study because the diary instrument was implemented after the initial polysomnogram; only the PSG-night rating was available at that time (Supplementary Table S1). Because V3 occurred without PAP and showed pronounced arousal-driven REM fragmentation with a higher proportion of excluded REM sleep, the mid-series dip in corrected RAI should be interpreted as context-dependent rather than as a stable biological reversal. Notably, when V3 is treated as a physiologically confounded visit,

corrected RAI increases monotonically from V1 to V2 to V4 (0.268 → 0.401 → 0.463), while tonic RSWA falls and phasic RSWA remains elevated.

Past medical history relevant to the present analysis included obstructive sleep apnoea treated with positive airway pressure (PAP). Melatonin exposure varied across follow-up, reflecting intermittent self-directed cessation and re-initiation; recorded doses at the time of V2–V4 were 2 mg, 6 mg, and 2 mg, respectively (Supplementary Table S1). He was not taking clonazepam, antidepressants (SSRIs/SNRIs/TCAs), antipsychotics, or dopaminergic agents during the peri-PSG windows. His neurologic examination was normal and he did not develop overt parkinsonism during the investigative period. The patient reported that dream enactment behaviours were most disruptive during periods of fragmented sleep, and that perceived night-to-night variability made it difficult to judge whether symptoms were improving. They valued objective PSG feedback but found that a single study did not always reflect their typical weeks.

Four attended vPSGs (V1–V4) were available over approximately 6.5 years. Standard sleep staging and respiratory scoring followed American Academy of Sleep Medicine criteria(Berry et al., 2012), and macrostructure/respiratory indices are summarised in Supplementary Table S1. Sleep efficiency varied across visits, with the lowest efficiency at V2 (60.1%) and otherwise broadly comparable values at V1, V3, and V4 (76.0%, 76.5%, 75.9%). REM proportion was lowest at V2 (14.9% of total sleep time) and higher at V1, V3, and V4 (23.3%, 16.6%, and 25.0%, respectively) (Supplementary Table S1). Arousal burden was notably increased at V3 (arousal index 36.2/h) relative to the other visits (15.0/h, 9.5/h, 12.7/h) (Supplementary Table S1). The V3 recording was inadvertently performed without PAP owing to a misunderstanding, coinciding with a recorded apnoea–hypopnoea index (20.5/h) and marked REM sleep instability, reflected by an increased number of REM bouts (n=46) (Supplementary Table S1). This visit therefore represents a clinically interpretable but confounded physiological context in which arousal-driven REM sleep fragmentation was prominent.

Across visits, quantitative REM atonia and RSWA components showed non-monotonic trajectories. Corrected RAI values were 0.268 (V1), 0.401 (V2), 0.313 (V3), and 0.463 (V4) (Supplementary Table S1). The tonic and phasic components were partially dissociated: tonic chin activity declined overall across the series (66.0%, 37.6%, 55.7%, 25.7%), whereas phasic chin activity rose from baseline and remained elevated (1.5%, 8.5%, 8.1%, 9.1%) (Supplementary Table S1; Figure 1). The proportion of REM sleep excluded from quantitative analysis due to artefact/contamination was minimal at V1–V2 (0%) and V4 (0.2%), but higher at V3 (14.3%), consistent with the more fragmented and arousal-rich recording (Supplementary Table S1). REM sleep minutes contributing to quantitative analysis were 76.5, 42.5, 65.7, and 74.4 minutes at V1–V4, respectively (Supplementary Table S1), providing context for interpretation of longitudinal RSWA estimates and for the “REM sleep opportunity” concept illustrated in Figure 2.

Clinically, diary-derived enactment burden was broadly stable from V2 to V4 (total enactment burden 2.4, 2.0, and 2.1 on a 0–5 scale), while harm severity remained 2/4 at V1–V3 but fell to 0/4 at V4 (Supplementary Table S1). These diary-derived ratings are presented as within-person descriptive measures rather than as validated severity outcomes. Notably, no sleep-related harm was reported at V4, which coincided with the highest corrected RAI and persistent high phasic

RSWA, emphasising that behavioural harm and quantitative RSWA are related but not interchangeable constructs in longitudinal assessment (Figure 1; Figure 2; Supplementary Table S1).

Discussion

RSWA is the electrophysiological hallmark required for the diagnosis of RBD, yet it is not a single physiological quantity. On contemporary definitions, RSWA is expressed as sustained (tonic) elevation of EMG activity during REM sleep and/or excessive transient (phasic) bursts, typically quantified in submental EMG with optional limb channels (Puligheddu et al., 2023). The practical problem for biomarker work is that tonic and phasic RSWA may index partly separable elements of REM motor dyscontrol, and the linkage between RSWA magnitude and overt dream enactment behaviour (DEB) is neither direct nor stable across disorders, stages, montages, or nights.

RSWA morphology and dream enactment: what is established

At the level of event generation, the best evidence indicates that complex motor–behavioural episodes in RBD occur disproportionately during phasic REM sleep periods, rather than during tonic REM sleep. In a structured vPSG analysis, the more complex episodes were enriched for rapid eye movements and transient EEG phenomena (including alpha bursts and sawtooth waves), consistent with a state of increased REM sleep microstructural activation immediately preceding enactment (Manni et al., 2009). This view is convergent with work emphasising the relation between abnormal behaviours and REM sleep microstructure, in which behaviours cluster with phasic REM sleep features (Frauscher et al., 2009). More recent EEG work in iRBD has identified reproducible cortical dynamics in the period preceding DEBs, supporting the notion that cortical state and REM sleep microstructure contribute materially to whether motor disinhibition becomes overt behaviour (Date et al., 2024).

At the level of signal detection, the SINBAR programme is relevant because it ties phasic muscle activity to the practical capture of clinical manifestations. The SINBAR montage (mentalis, flexor digitorum superficialis, extensor digitorum brevis) was developed empirically to provide normative EMG values and optimise diagnostic discrimination (Frauscher et al., 2012). In subsequent work, simultaneous recording of these muscles detected the large majority of motor and vocal manifestations, and increased phasic EMG was proposed as an efficient trigger for targeted video review (Iranzo et al., 2011). These observations are often read as indirect support for the primacy of phasic activity in behavioural expression.

Two limits should be stated. First, these studies speak principally to the timing of episodes and the efficiency of detection, not to the biological meaning of RSWA as a marker of neurodegenerative burden. Secondly, they do not establish a monotone relationship between phasic RSWA magnitude and the severity or injuriousness of DEB; the episode-level literature supports phasic REM as the favoured context for enactment. However, the clinical severity of enactment likely reflects additional factors, many of which are not measured by chin EMG metrics alone.

RSWA as a biomarker of underlying pathology

When RSWA is used as a biomarker, either for phenoconversion risk in iRBD or for staging within established synucleinopathy, the literature is more heterogenous. In a large iRBD cohort study, tonic REM sleep muscle activity at baseline was the strongest predictor of phenoconversion risk (Singh et al., 2023). Independent longitudinal work has likewise supported RSWA severity as a risk-stratification marker, though with methodological heterogeneity in the definition of RSWA (McCarter et al., 2019). A contemporary review of predictors of progression notes that tonic and phasic RSWA may reflect different pathophysiological alterations within brainstem REM circuitry and summarises the emerging but not yet settled evidence on their differential predictive roles (de Natale et al., 2022).

A separate, clinically important observation is that RSWA and subjective DEB severity can decouple in established Parkinson disease. In a longitudinal vPSG study, approximately one quarter of patients reported subjective improvement of RBD symptoms, whilst all RSWA measures increased significantly at follow-up (Figorilli et al., 2020). This result does not prove that DEB disappears whilst RSWA progresses in every patient. However, it does provide a concrete precedent for the scenario that clinicians recognise, clinical expression may attenuate without parallel reduction in RSWA.

The interpretive consequence is that the field should be cautious about treating any single RSWA summary measure as a complete representation of the state of REM sleep motor control. This is also relevant for atonia indices such as the REM Atonia Index/RAI, which were designed to quantify the proportion of REM time spent in low-amplitude submental EMG and have strong validation for diagnostic use (Figorilli et al., 2017, Ferri et al., 2010, Ferri et al., 2008). In principle, an atonia index can improve if sustained background tone diminishes, even if short phasic bursts persist or increase, because those bursts occupy a small fraction of REM time. In this case corrected RAI improved at the final timepoint whilst phasic chin activity remained high (Fig. 1; Table 2), a pattern that is physiologically plausible within the framework and clinically consequential for how longitudinal improvement is interpreted.

Two brief clinical modifiers are also worth noting. Intermittent melatonin exposure in RBD has been suggested to preferentially reduce tonic manifestations of RSWA, and may thereby theoretically exaggerate tonic–phasic dissociation in some patients(Takeuchi et al., 2001, Kunz and Mahlberg, 2010). Similarly, the V3 “dip” in corrected RAI occurred on a night with PAP omission, high arousal burden, and heavy REM sleep fragmentation. Given evidence that the elevated EMG milieu of RBD can interact with OSA severity and REM sleep physiology, respiratory instability should be treated as a first-order confounder when interpreting apparent longitudinal change in RSWA indices(Huang et al., 2011).

iRBD, phenoconverted RBD, and RSWA without RBD: stage and aetiology matter

RBD in mid-to-late life, when confirmed on vPSG, is strongly associated with prodromal α-synucleinopathy, and this framing has been formalised in high-level reviews and biomarker-oriented position pieces (Miglis et al., 2021, Högl et al., 2018, Dauvilliers et al., 2018). Yet two complications are now well documented.

First, RSWA can occur without clinical RBD, particularly in early-stage Parkinson disease. A recent Movement Disorders analysis emphasised that isolated RSWA is not equivalent to RBD in early PD, reinforcing that the electrophysiological substrate may be present without manifest enactment behaviour (Dodet et al., 2024). This is directly germane to the question of disappearing re-enactments. The absence of overt episodes does not, by itself, adjudicate the status of the REM sleep atonia network.

Secondly, RSWA is not exclusive to synucleinopathy. In probable Alzheimer disease, one polysomnographic series concluded that RBD was rare, but RSWA was relatively frequent (Gagnon et al., 2006). The biological interpretation remains uncertain but it nevertheless cautions against assuming that RSWA morphology always maps to the same upstream lesion across disorders.

Medication-associated RSWA provides a further example of aetiological heterogeneity. Serotonergic antidepressants have been associated with increased submental EMG tone during REM sleep, with some work suggesting a preferential effect on tonic rather than phasic components (Winkelman and James, 2004). In a large clinical cohort, antidepressant treatment was associated with elevated RSWA without necessarily meeting full RBD criteria. Importantly, differences in RSWA distribution/type were observed between antidepressant-treated patients and Parkinson disease-RBD patients, with relatively greater anterior tibialis RSWA in the former and higher tonic RSWA in the latter (McCarter et al., 2015). Thus, RSWA likely reflects multiple mechanisms, and the muscles sampled may determine which is most apparent.

Why the field still lacks a settled RSWA biomarker

The dominance of chin EMG in routine practice is pragmatic, but it is not necessarily optimal for biomarker inference. SINBAR-derived work demonstrates that combining mentalis with limb muscles increases the capture of phasic activity and improves linkage to behavioural manifestations (Frauscher et al., 2012, Iranzo et al., 2011). Software implementations of SINBAR criteria continue to operationalise this multi-muscle approach (Röthenbacher et al., 2022) and recent work suggests that certain phasic limb metrics show excellent discriminative performance(Frauscher et al., 2012, Cesari et al., 2021).

What remains poorly established is whether different muscle groups and RSWA morphologies exhibit different temporal trajectories across the prodrome and after phenoconversion. If tonic and phasic components can diverge within a single muscle (Fig. 1), it is also plausible that divergence may be magnified across muscle groups, given that cranial and limb musculature are governed by partially distinct motor pools and may be differentially influenced by brainstem and spinal mechanisms. The field presently has limited longitudinal multi-muscle data with stable montage and transparent artefact handling sufficient to resolve this.

Where this case sits and what it adds

Three observations from this case can be similarly compared against the state of knowledge. Firstly, atonia indices can improve whilst phasic RSWA persists. This is consistent with what an atonia index measures (Ferri et al., 2010, Ferri et al., 2008). It follows that improvement in a global atonia

metric does not imply resolution of phasic disinhibition, and component-wise reporting is not optional if one wishes to interpret directionality (Fig. 1; Table 2). Secondly, PSG-night DEB capture is constrained by REM opportunity. In this patient, PSG-night enactment frequency tracked REM sleep duration far more closely than it tracked tonic/phasic/RAI measures (Supplementary Table S1; Fig. 2B). This sits comfortably with the episode-level literature locating DEB in phasic REM sleep microstructure (Manni et al., 2009) and with the broader recognition that vPSG samples a limited and state-dependent portion of a variable phenotype. And finally, this case illustrates the DEB–RSWA dissociation problem. Longitudinal PD data demonstrate that subjective DEB improvement can coexist with increasing RSWA (Figorilli et al., 2020). The present case adds a mechanistically coherent variant: a global atonia metric improved while phasic activity persisted, and behavioural sampling varied across peri-PSG windows.

When dream re-enactment attenuates, whether by progression, adaptation, or state change, does RSWA attenuate, remain stable, or redistribute across tonic/phasic and across muscle groups? The answer remains unknown but a parsimonious explanation for such dissociation could be that the determinants of RSWA and the determinants of overt enactment are not identical. A further possibility, necessarily speculative, is that the dissociation may reflect variation in suprapontine gating rather than variation in the brainstem substrate alone. Motor–behavioural episodes in RBD occur preferentially during phasic REM sleep and frequently coincide with phasic EEG/EOG phenomena, consistent with transient cortical activation immediately upstream of enactment (Manni et al., 2009, Date et al., 2024). In Parkinson disease, subjective attenuation of RBD behaviours has been reported despite increasing RSWA on follow-up, implying that behavioural expression can diminish without recovery of REM atonia (Figorilli et al., 2020). Recent semiological work comparing iRBD with narcolepsy-related RBD, a context of orexin deficiency, demonstrates categorical differences in motor topography and complexity, supporting the broader proposition that neuromodulatory milieu and suprapontine integration may shape which motor patterns are released when REM atonia fails (Drakatos et al., 2025). Under such a scheme, longitudinal change in RSWA may not be mirrored by longitudinal change in DEB, and muscle-specific trajectories may differ by aetiology. This position remains hypothetical and its value is that it makes testable predictions about measurement. For example, if enactment is preferentially released during particular REM sleep microstates, then component-wise RSWA should be reported alongside REM sleep microstructure and phasic EEG/EOG context.

Several uncertainties follow from the present state of evidence and known unknowns include the extent to which tonic versus phasic RSWA differentially predicts phenoconversion across cohorts (Singh et al., 2023, de Natale et al., 2022); whether RSWA trajectories differ before and after phenoconversion; and whether muscle-group involvement shifts systematically with disease stage or aetiology (Frauscher et al., 2012, McCarter et al., 2015). A plausible class of unrecognised determinants concerns REM sleep microstructure and cortical state. Episode-level evidence implicates phasic REM sleep features and transient EEG phenomena in the immediate prelude to DEB (Manni et al., 2009, Date et al., 2024). Yet most RSWA biomarker work remains anchored to EMG summaries. It is therefore possible that part of the apparent inconsistency across cohorts reflects a mismatch between what is quantified (global EMG summaries) and what precipitates behaviour (a conjunction of disinhibition with REM sleep microstructure and cortical activation).

The case supports a restrained methodological position. Longitudinal RSWA reporting intended to inform biomarker inference should, at minimum, present tonic and phasic measures alongside an atonia index, and should state the montage, the amount of REM sleep analysed, and the fraction excluded for contamination (Puligheddu et al., 2023). Where feasible, stable multi-muscle recording informed by SINBAR experience should be adopted, since it improves capture of phasic activity linked to behavioural manifestations and may reveal differential muscle trajectories (Frauscher et al., 2012, Iranzo et al., 2011, Röthenbacher et al., 2022). Finally, if the aim is to relate RSWA to behavioural severity, then peri-PSG behavioural sampling and REM sleep microstructure metrics ought to be treated as first-order variables rather than as incidental context.

Take-home message

In conclusion, in this case report, using a transparent longitudinal series, we demonstrate that RSWA components and behavioural expression can separate in time and direction, and that this separation is consistent with established event-level physiology and with longitudinal observations in Parkinson disease. Moreover, we advance that these findings may suggest that RSWA should be treated as a composite phenotype whose meaning is contingent upon component, muscle, state, and stage.

Conflict of Interest

The authors declare that the research was conducted in the absence of any commercial or financial relationships that could be construed as a potential conflict of interest.

Funding

This work was funded in whole, or in part, by the Wellcome Trust (103952/Z/14/Z). This work also represents independent research supported in part by the NIHR Maudsley Biomedical Research Centre at South London and Maudsley NHS Foundation Trust and King's College London.

Data Availability

The original contributions presented in the study are included in the article/Supplementary material, further inquiries can be directed to the corresponding author.

Ethics Statement

Ethical approval was not required for this case report as it involves a single patient case study with informed written consent obtained from the patient. The studies were conducted in accordance with the local legislation and institutional requirements. The participants provided their written informed consent to participate in this study. Written informed consent was obtained from the individual(s) for the publication of any potentially identifiable images or data included in this article.

Generative AI statement

The author(s) declare that no Gen AI was used in the creation of this manuscript.

Author Contributions (CRediT)

Conceptualization: I.R.; Methodology: N.B., I.P., C.S., I.R.; Investigation/Clinical assessment: V.G., A.B., S.D., D.O'R., I.R.; Data curation: V.G., I.R., A.B., S.D., D.O'R.; Formal analysis: N.B., I.P., C.S.; Visualization: N.B., I.P., C.S.; Writing – original draft: V.G., I.P, N.B. C.S., D.O'R., I.R.; Writing – review & editing: all authors; Supervision: I.R.

Acknowledgments

We thank colleagues at the Sleep Disorders Centre, Guy's and St Thomas' NHS Foundation Trust, for their support. We specifically thank the patient for their generous time and engagement with the study.

References

Date, S., Sumi, Y., Fujiwara, K., Imai, M., Ogawa, K., & Kadotani, H. (2024). Polysomnographic features prior to dream enactment behaviors in isolated rapid eye movement sleep behavior disorder. *Clinical Neurophysiology*, *166*, 74-86. https://doi.org/10.1016/j.clinph.2024.07.013

Dauvilliers, Y., Schenck, C. H., Postuma, R. B., Iranzo, A., Luppi, P. H., Plazzi, G., Montplaisir, J., & Boeve, B. (2018). REM sleep behaviour disorder. *Nature Reviews Disease Primers*, *4*(1), 19. https://doi.org/10.1038/s41572-018-0016-5

de Natale, E. R., Wilson, H., & Politis, M. (2022). Predictors of RBD progression and conversion to synucleinopathies. *Current Neurology and Neuroscience Reports*, *22*(2), 93-104. https://doi.org/10.1007/s11910-022-01171-0

Dodet, P., Houot, M., Leu-Semenescu, S., Gaurav, R., Mangone, G., Corvol, J. C., Lehéricy, S., Vidailhet, M., Roze, E., & Arnulf, I. (2024). Isolated REM sleep without atonia is not equivalent to REM sleep behavior disorder in early-stage Parkinson's disease. *Movement Disorders*, *39*(7), 1190-1202. https://doi.org/10.1002/mds.29813

Drakatos, P., Duncan, I., Wasserman, D., Gnoni, V., O'Regan, D., Varga, A. W., Tan, H., Ashkan, K., Grinberg, L. T., Parrino, L., Ferini-Strambi, L., Goadsby, P. J., Chaudhuri, K. R., & Rosenzweig, I. (2025). Differential motor signatures in isolated and narcolepsy-related REM sleep behaviour disorder: A preliminary study. *Frontiers in Neurology*, *16*, 1749306. https://doi.org/10.3389/fneur.2025.1749306

Ferri, R., Manconi, M., Plazzi, G., Bruni, O., Vandi, S., Montagna, P., Ferini-Strambi, L., & Zucconi, M. (2008). A quantitative statistical analysis of the submentalis muscle EMG amplitude during sleep in normal controls and patients with REM sleep behavior disorder. *Journal of Sleep Research*, *17*(1), 89-100. https://doi.org/10.1111/j.1365-2869.2008.00631.x

Ferri, R., Marelli, S., Cosentino, F. I., Rundo, F., Ferini-Strambi, L., & Zucconi, M. (2013). Night-to-night variability of automatic quantitative parameters of the chin EMG amplitude (Atonia Index) in REM sleep behavior disorder. *Journal of Clinical Sleep Medicine*, *9*(3), 253-258. https://doi.org/10.5664/jcsm.2490

Ferri, R., Rundo, F., Manconi, M., Plazzi, G., Bruni, O., Oldani, A., Ferini-Strambi, L., & Zucconi, M. (2010). Improved computation of the atonia index in normal controls and patients with REM sleep behavior disorder. *Sleep Medicine*, *11*(9), 947-949. https://doi.org/10.1016/j.sleep.2010.06.003

Figorilli, M., Ferri, R., Zibetti, M., Beudin, P., Puligheddu, M., Lopiano, L., Cicolin, A., Durif, F., Marques, A., & Fantini, M. L. (2017). Comparison between automatic and visual scorings of REM sleep without atonia for the diagnosis of REM sleep behavior disorder in Parkinson disease. *Sleep*, *40*(2), zsw060. https://doi.org/10.1093/sleep/zsw060

Figorilli, M., Marques, A. R., Vidal, T., Delaby, L., Meloni, M., Pereira, B., Lambert, C., Puligheddu, M., Durif, F., & Fantini, M. L. (2020). Does REM sleep behavior disorder change in the progression of Parkinson's disease? *Sleep Medicine*, *68*, 190-198. https://doi.org/10.1016/j.sleep.2019.12.013

Frauscher, B., Gschliesser, V., Brandauer, E., Ulmer, H., Poewe, W., & Högl, B. (2009). The relation between abnormal behaviors and REM sleep microstructure in patients with REM sleep behavior disorder. *Sleep Medicine*, *10*(2), 174-181. https://doi.org/10.1016/j.sleep.2008.01.003

Frauscher, B., Iranzo, A., Gaig, C., Gschliesser, V., Guaita, M., Raffelseder, V., Ehrmann, L., Sola, N., Salamero, M., Tolosa, E., Poewe, W., Santamaria, J., & Högl, B. (2012). Normative EMG values during REM sleep for the diagnosis of REM sleep behavior disorder. *Sleep*, *35*(6), 835-847. https://doi.org/10.5665/sleep.1886

Gagnon, J. F., Petit, D., Fantini, M. L., Rompré, S., Gauthier, S., Panisset, M., Robillard, A., & Montplaisir, J. (2006). REM sleep behavior disorder and REM sleep without atonia in probable Alzheimer disease. *Sleep*, *29*(10), 1321-1325. https://doi.org/10.1093/sleep/29.10.1321

Högl, B., Stefani, A., & Videnovic, A. (2018). Idiopathic REM sleep behaviour disorder and neurodegeneration - an update. *Nature Reviews Neurology*, *14*(1), 40-55. https://doi.org/10.1038/nrneurol.2017.157

Iranzo, A., Fernández-Arcos, A., Tolosa, E., Serradell, M., Molinuevo, J. L., Valldeoriola, F., Gelpi, E., Vilaseca, I., Sánchez-Valle, R., Lladó, A., Gaig, C., & Santamaría, J. (2014). Neurodegenerative disorder risk in idiopathic REM sleep behavior disorder: study in 174 patients. *PLoS One*, *9*(2), e89741. https://doi.org/10.1371/journal.pone.0089741

Iranzo, A., Frauscher, B., Santos, H., Gschliesser, V., Ratti, L., Falkenstetter, T., Stürner, C., Salamero, M., Tolosa, E., Poewe, W., Santamaria, J., & Högl, B. (2011). Usefulness of the SINBAR electromyographic montage to detect the motor and vocal manifestations occurring in REM sleep behavior disorder. *Sleep Medicine*, *12*(3), 284-288. https://doi.org/10.1016/j.sleep.2010.04.021

Iranzo, A., Ratti, P. L., Casanova-Molla, J., Serradell, M., Vilaseca, I., & Santamaria, J. (2009). Excessive muscle activity increases over time in idiopathic REM sleep behavior disorder. *Sleep*, *32*(9), 1149-1153. https://doi.org/10.1093/sleep/32.9.1149

Iranzo, A., Santamaria, J., & Tolosa, E. (2016). Idiopathic rapid eye movement sleep behaviour disorder: diagnosis, management, and the need for neuroprotective interventions. *The Lancet Neurology*, *15*(4), 405-419. https://doi.org/10.1016/s1474-4422(16)00057-0

Manni, R., Terzaghi, M., & Glorioso, M. (2009). Motor-behavioral episodes in REM sleep behavior disorder and phasic events during REM sleep. *Sleep*, *32*(2), 241-245. https://doi.org/10.1093/sleep/32.2.241

McCarter, S. J., Sandness, D. J., McCarter, A. R., Feemster, J. C., Teigen, L. N., Timm, P. C., Boeve, B. F., Silber, M. H., & St Louis, E. K. (2019). REM sleep muscle activity in idiopathic REM sleep behavior disorder predicts phenoconversion. *Neurology*, *93*(12), e1171-e1179. https://doi.org/10.1212/wnl.0000000000008127

McCarter, S. J., St Louis, E. K., Sandness, D. J., Arndt, K., Erickson, M., Tabatabai, G., Boeve, B. F., & Silber, M. H. (2015). Antidepressants Increase REM Sleep Muscle Tone in Patients with and without REM Sleep Behavior Disorder. *Sleep*, *38*(6), 907-917. https://doi.org/10.5665/sleep.4738

Miglis, M. G., Adler, C. H., Antelmi, E., Arnaldi, D., Baldelli, L., Boeve, B. F., Cesari, M., Dall'Antonia, I., Diederich, N. J., Doppler, K., Dušek, P., Ferri, R., Gagnon, J. F., Gan-Or, Z., Hermann, W., Högl, B., Hu, M. T., Iranzo, A., Janzen, A.,…Oertel, W. H. (2021). Biomarkers of conversion to α-synucleinopathy in isolated rapid-eye-movement sleep behaviour disorder. *The Lancet Neurology*, *20*(8), 671-684. https://doi.org/10.1016/s1474-4422(21)00176-9

Puligheddu, M., Figorilli, M., Congiu, P., Lecca, R., Casaglia, E., Tamburrino, L., Orrù, R., Meloni, F., & Ferri, R. (2023). Quantification of REM sleep without atonia: A review of study methods and meta-analysis of their performance for the diagnosis of RBD. *Sleep Medicine Reviews*, *68*, 101745. https://doi.org/10.1016/j.smrv.2023.101745

Roguski, A., Rayment, D., Whone, A. L., Jones, M. W., & Rolinski, M. (2020). A Neurologist's Guide to REM Sleep Behavior Disorder. *Frontiers in Neurology*, *11*, 610. https://doi.org/10.3389/fneur.2020.00610

Röthenbacher, A., Cesari, M., Doppler, C. E. J., Okkels, N., Willemsen, N., Sembowski, N., Seger, A., Lindner, M., Brune, C., Stefani, A., Högl, B., Bialonski, S., Borghammer, P., Fink, G. R., Schober, M., & Sommerauer, M. (2022). RBDtector: an open-source software to detect REM sleep without atonia according to visual scoring criteria. *Scientific Reports*, *12*(1), 20886. https://doi.org/10.1038/s41598-022-25163-9

Singh, A., Williams, S., Calabrese, A., & Riha, R. (2023). Tonic REM sleep muscle activity is the strongest predictor of phenoconversion risk to neurodegenerative disease in isolated REM sleep behaviour disorder. *Journal of Sleep Research*, *32*(3), e13792. https://doi.org/10.1111/jsr.13792

Winkelman, J. W., & James, L. (2004). Serotonergic antidepressants are associated with REM sleep without atonia. *Sleep*, *27*(2), 317-321. https://doi.org/10.1093/sleep/27.2.317

Figure legends

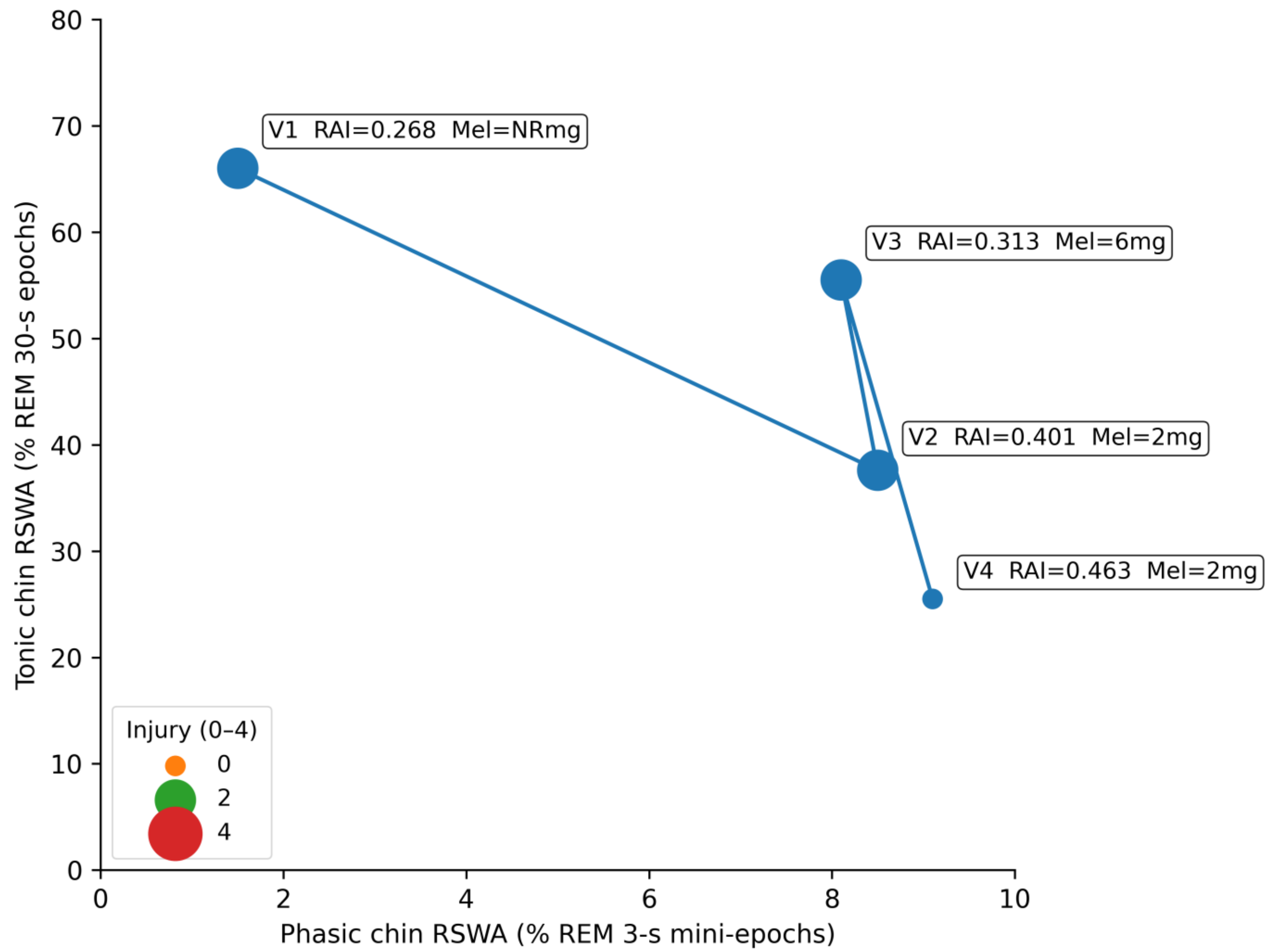

Figure 1. Longitudinal shift in submental RSWA phenotype in tonic–phasic space, with concurrent sleep-related inadvertent harm severity. Each point represents one attended overnight video-polysomnogram (vPSG) (V1–V4) plotted in a phase plane defined by phasic (x-axis) and tonic (y-axis) submental REM sleep without atonia (RSWA). Phasic submental RSWA is expressed as the percentage of analysed REM sleep 3-second mini-epochs containing ≥1 phasic EMG burst. Tonic submental RSWA is expressed as the percentage of analysed REM sleep 30-second epochs meeting tonic criteria. Points are connected in chronological order to indicate the direction of follow-up (time spacing not to scale). Marker area is proportional to sleep-related harm severity (ordinal diary-derived score, 0–4) anchored to each vPSG (inset size key). Each callout

lists the visit identifier, corrected REM atonia index (RAI; 0–1), and melatonin dose (mg) at the time of the study, facilitating cross-reference to Table 2 and Supplementary Table S1. Quantification was performed using an identical longitudinal submental EMG extraction pipeline across visits; REM segments excluded because of non-physiological artefact or physiological contamination (including arousal-linked activation and respiratory-event adjacency) were removed prior to computation (see Table 2 and Supplementary Table S1).

*Abbreviations***:** EMG, electromyography; RAI, REM atonia index; REM, rapid eye movement; RSWA, REM sleep without atonia; vPSG, video-polysomnography.

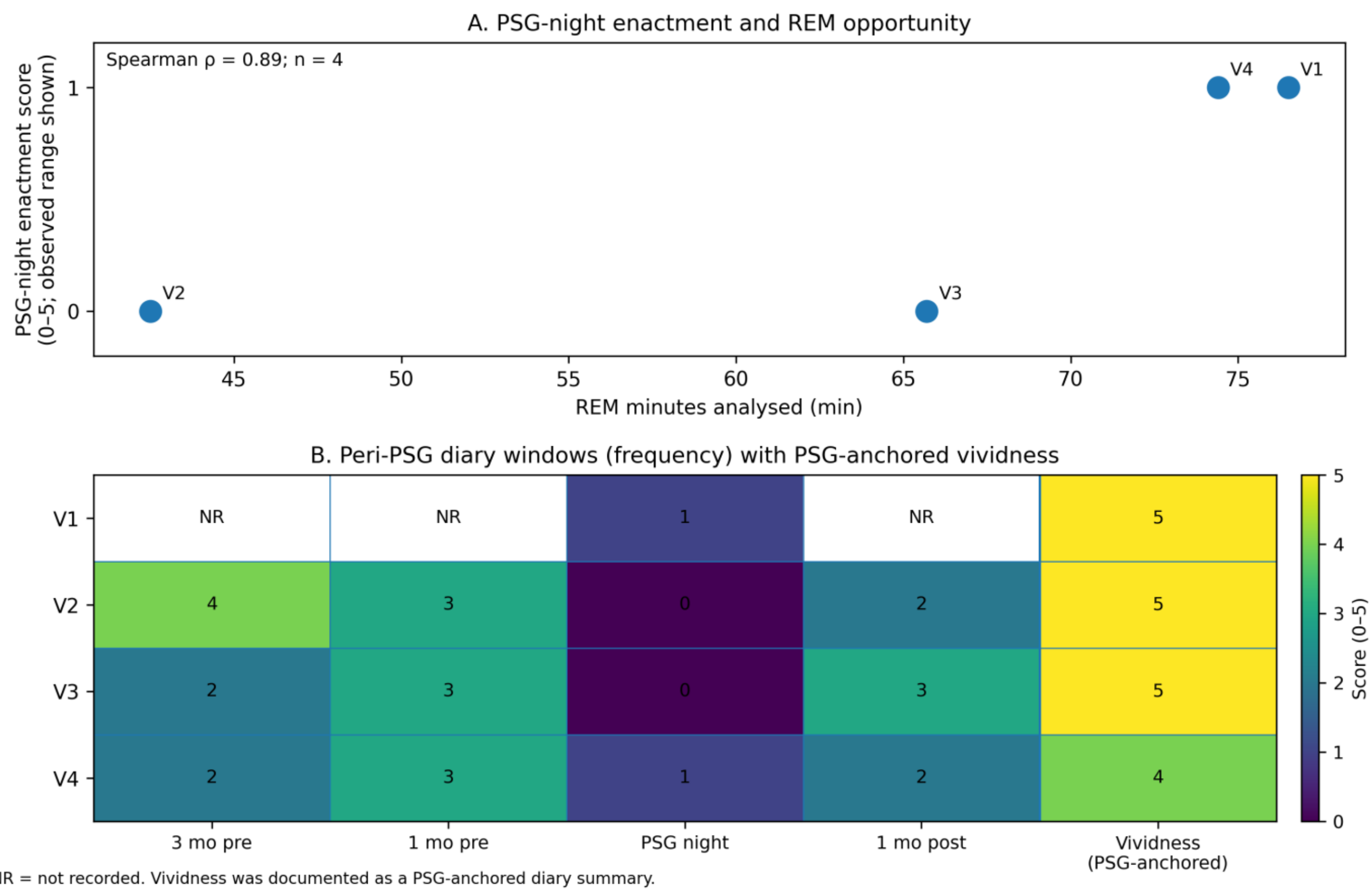

Figure 2. REM opportunity and peri-PSG symptom sampling. (A) PSG-night dream enactment frequency (F_PSG, ordinal 0–5 scale) plotted against REM minutes analysed for RSWA/RAI quantification across four attended video-polysomnograms (V1–V4). Each point represents one vPSG and is labelled by visit. The Spearman rank correlation coefficient (ρ) and *n* are shown; no p-value is presented. For legibility, the y-axis is restricted to the observed PSG-night scores in this case (0–1); the underlying diary scale is 0–5. (B) Diary matrix summarising peri-PSG behavioural sampling. Rows correspond to

visits (V1–V4). Columns report diary-derived enactment frequency (0–5) for four windows anchored to each vPSG (3 months pre-vPSG, 1 month pre-vPSG, vPSG night, and 1 month post-vPSG), plus a final column showing dream vividness (0–5) recorded as a PSG-anchored diary summary (not time-resolved across windows). Cell shading encodes the raw ordinal score (0–5), with the numeric value printed within each cell; NR denotes not recorded (diary not instituted until after V1 for the pre/post windows). The vertical divider separates the time-resolved peri-PSG windows from the PSG-anchored vividness measure.

Abbreviations: F_PSG, PSG-night enactment frequency score; NR, not recorded; PSG, polysomnogram; REM, rapid eye movement; RSWA, REM sleep without atonia; vPSG, video-polysomnography

Table 1. Selected longitudinal and methodological literature relevant to RSWA quantification in iRBD.

Citation	Design / sample	Muscles / montage	RSWA metric(s)	Key message
**Iranzo et al. (2009)**	Idiopathic RBD; longitudinal PSG follow-up	Study-specific chin/limb EMG	Tonic/phasic EMG activity	Group-level RSWA increases reported; does not entail monotonic within-individual trajectories.
**Ferri et al. (2010)**	Methodological development	Submental EMG	Atonia index / RAI framework	Automated atonia metrics depend on preprocessing and signal quality; artefact handling is central.
**Ferri et al. (2013)**	Night-to-night variability	Submental EMG	Atonia index and activation measures	Atonia index shows modest variability; activation measures vary more, supporting component-wise reporting.
**Frauscher et al. (2012)**	Normative EMG values; SINBAR montage	Multi-muscle montage (SINBAR)	Tonic and phasic thresholds	Montage selection materially influences RSWA detection; limb muscles add information beyond chin alone.

Abbreviations: iRBD, isolated REM sleep behaviour disorder; PSG, polysomnogram; RAI, REM atonia index; REM, rapid eye movement; RSWA, REM sleep without atonia; SINBAR, Sleep Innsbruck Barcelona montage.

Table 2. RSWA pipeline transparency across visits (submental-anchored analysis).

Parameter	V1	V2	V3	V4
**REM sleep minutes analysed (min)**	76.5	42.5	65.7	74.4
**REM sleep excluded due to artefact/contamination (%)**	0	0	14.3	0.2
**Number of REM bouts**	7	9	46	31
**REM atonia index, corrected (RAI)**	0.268	0.401	0.313	0.463

Notes: 'REM sleep excluded' reflects REM sleep mini-epochs removed by pre-specified exclusion rules for artefact and physiologic contamination. REM sleep bouts count provides a descriptive index of REM sleep fragmentation across the night (bout = contiguous REM sleep separated by non-REM or wake).

SUPPLEMENT

Expanded Methods and Materials

Design and consent: This longitudinal case report is derived from clinical-care, attended video-polysomnography (vPSG) performed on four occasions (V1–V4) and from diary-anchored interviews conducted around each vPSG. Written informed consent for publication was obtained. Dates are coarsened to preserve anonymisation.

*Clinical characterisation: S*ymptoms were characterised using a structured interview anchored to each vPSG. Diary-derived enactment frequency ratings (0–5) were obtained for four temporal windows: 3 months pre-PSG, 1 month pre-PSG, the PSG night, and 1 month post-PSG. Dream vividness (0–5), dream recall frequency (0–4), and sleep-related harm severity (0–4) were coded according to a pre-specified interview guide. These ordinal diary ratings and the composite enactment burden were used for within-person tracking and are not intended as validated severity scales. Where a domain was recorded only as a PSG-anchored diary summary rather than per window, the PSG-anchored value was carried across windows for descriptive purposes. For treatment exposure relevant to REM sleep motor expression, melatonin dose at the time of each study was recorded (Supplementary Table S1).

Polysomnography acquisition and scoring: All studies were attended overnight vPSG with synchronised audio-video and standard channels including EEG, EOG, ECG, and submental EMG, with respiratory signals scored according to contemporaneous American Academy of Sleep Medicine criteria. Owing to technical discontinuity, limb EMG channels were not comparable across all visits and were not used for longitudinal inference. Longitudinal RSWA inference was therefore anchored to submental EMG, which was technically interpretable at all visits.

RSWA quantification and REM accounting: Quantitative REM muscle tone was characterised using three complementary submental EMG metrics: a REM atonia index (RAI) and component measures of tonic and phasic RSWA. RAI was computed within REM sleep using 3-second mini-epochs classified as atonia, intermediate, or non-atonia; the index was calculated as:

$$\mathrm{RAI} = \frac{N_{\mathrm{atonia}} + 0.5 \cdot N_{\mathrm{intermediate}}}{N_{\mathrm{total\ mini\text{-}epochs}}}$$

A “corrected” RAI was derived after pre-specified exclusion of REM mini-epochs contaminated by non-physiological artefact or by physiological contamination likely to inflate EMG amplitude unrelated to intrinsic RSWA (including arousal-linked activation

and respiratory-event adjacency). For transparency, the REM sleep minutes contributing to analysis, the proportion of REM sleep excluded, and the number of REM bouts (contiguous REM sleep segments separated by non-REM sleep or wake) are reported for each visit (Supplementary Table S1). REM bout counts are sensitive to scoring of brief stage intrusions and arousals and are therefore presented descriptively.

Submental EMG was band-pass filtered (10–100 Hz), rectified, and smoothed with a 100-ms moving window. Activity was evaluated in 3-s mini-epochs using visit-specific thresholds: T_{low} was the 20th percentile of mini-epoch median amplitudes in REM sleep (artifact-cleaned), $T_{high} = 2 \times T_{low}$; tonic RSWA was defined when ≥50% of a 30-s REM epoch exceeded T_{high}, and phasic RSWA as 0.1–5 s bursts exceeding $4 \times T_{low}$ within a mini-epoch, with hysteresis thresholds of 1.8× (onset) and 1.2× (offset) to reduce rapid switching. For the corrected RAI, REM mini-epochs within ±10 s (anchored to event onset) of any scored respiratory event (apnoea, hypopnoea, RERA) or AASM-defined arousal were excluded.

Tonic and phasic RSWA components: Tonic submental activity was expressed as the percentage of REM 30-second epochs meeting tonic criteria (denominator: analysed REM 30-second epochs). Phasic activity was expressed as the percentage of REM 3-second mini-epochs containing phasic bursts (denominator: analysed REM 3-second mini-epochs). These were analysed alongside corrected RAI to characterise longitudinal change and potential tonic–phasic dissociation (Figure 1).

Exploratory association analysis: Given the small number of longitudinal observations (n=4), inferential modelling was not pursued. Pre-specified exploratory associations were examined descriptively using Spearman rank correlations between selected diary-derived measures (PSG-night enactment frequency; sleep-related harm severity) and (i) REM opportunity (REM minutes analysed) and (ii) RSWA components (corrected RAI, tonic and phasic submental activity). Correlation coefficients are reported with n (Supplementary Table S2); p-values were not emphasised.

Supplementary Table S0. Timeline of clinical context, REM sleep opportunity, and RSWA metrics across visits.

Visit	Timing (approx.)	PAP on study night	Melatonin (mg)	AHI (/h)	Arousal index (/h)	REM (% TST)	REM minutes analysed	REM excluded (%)	REM bouts	Corrected RAI	Tonic chin (%)	Phasic chin (%)	PSG-night enactment (0–5)	Sleep-related harm severity (0-4)

**V 1**	Baseline	Yes	NR	—	15.0	23.3	76.5	0.0	7	0.268	66.0	1.5	1	2
**V 2**	+ 1 year	Yes	2	—	9.5	14.9	42.5	0.0	9	0.401	37.6	8.5	0	2
**V 3**	+4 years	No (PAP omitted)	6	20.5	36.2	16.6	65.7	14.3	46	0.313	55.7	8.1	0	2
**V 4**	+1.5 years	Yes	2	—	12.7	25.0	74.4	0.2	31	0.463	25.7	9.1	1	0

Supplementary Table S1. Longitudinal clinical and polysomnographic measures across visits. Values are shown for each visit (V1–V4). Clinical diary measures refer to the specified peri-PSG window. PSG metrics are derived from attended video-polysomnography; REM sleep analysis uses mini-epoch scoring as described in Methods.

Parameter	V1	V2	V3	V4
**Melatonin dose (mg)**	NR	2	6	2
**Diary enactment frequency (3 mo pre; 0-5)**	NR	4	2	2
**Diary enactment frequency (1 mo pre; 0-5)**	NR	3	3	3
**Diary enactment frequency (PSG night; 0-5)**	1	0	0	1
**Diary enactment frequency (1 mo post; 0-5)**	NR	2	3	2
**Diary enactment burden (TEB; 0-5)**	NR	2.4	2	2.1
**Dream vividness (0-5)**	5	5	5	4
**Dream recall (0-4)**	4	4	4	4
**Sleep-related harm severity (0-4)**	2	2	2	0
**Composite enactment score (CES4D; 0-5)**	NR	3.7	3.6	2.8
**REM atonia index, corrected (RAI)**	0.268	0.401	0.313	0.463
**RSWA - tonic chin (% REM 30-s epochs)**	66	37.6	55.7	25.7
**RSWA - phasic chin (% REM 3-s mini-epochs)**	1.5	8.5	8.1	9.1
**REM sleep excluded due to artefact/contamination (%)**	0	0	14.3	0.2
**REM minutes analysed (min)**	76.5	42.5	65.7	74.4
**Number of REM bouts**	7	9	46	31
**Sleep efficiency (%)**	76	60.1	76.5	75.9
**Arousal index (/h)**	15	9.5	36.2	12.7
**N3 (% TST)**	25.7	32.1	10.2	17.3
**REM (% TST)**	23.3	14.9	16.6	25

Notes: Diary enactment frequency and burden are reported on 0–5 scales as defined in the manuscript; harm severity is reported on a 0–4 scale. TEB, total enactment burden. AHI, apnea–hypopnea index; ODI, oxygen desaturation index; PAP, positive airway pressure; RAI, REM atonia index. REM minutes analysed refers to REM time remaining after pre-specified exclusion of REM mini-epochs contaminated by artefact and physiologic activation (e.g., respiratory-event adjacency or arousal-related EMG), as detailed in Methods. Percent REM excluded is the proportion of REM mini-epochs removed from RAI/RSWA quantification under these rules.

Supplementary Table S2. Pre-specified rank associations between diary-derived measures and submental RSWA/REM sleep metrics.

Spearman's ρ is reported without p-values owing to the small number of repeated vPSG timepoints. n denotes the number of paired observations contributing to each estimate. Where an input variable is constant across visits, ρ is not defined and is reported as NA (constant).

Pre-specified association	Spearman ρ	n
**PSG-night enactment frequency (F_PSG) vs REM minutes analysed**	0.89	4
**PSG-night enactment frequency (F_PSG) vs corrected RAI**	0	4
**PSG-night enactment frequency (F_PSG) vs tonic chin activity (%)**	0	4
**PSG-night enactment frequency (F_PSG) vs phasic chin activity (%)**	0	4
**Sleep-related harm severity (0-4) vs tonic chin activity (%)**	0.77	4
**Sleep-related harm severity (0-4) vs corrected RAI**	-0.77	4
**Sleep-related harm severity (0-4) vs phasic chin activity (%)**	-0.77	4
**Vividness (0–5) vs corrected RAI**	-0.77	4
**Dream recall frequency (0–4) vs corrected RAI**	NA (constant)	4
**Frequency burden (F_TEB) vs corrected RAI**	0.5	3
**Frequency burden (F_TEB) vs phasic chin activity (%)**	0.5	3
**REM sleep (% TST) vs corrected RAI**	0.2	4

ARRIGONI, E., CHEN, M. C. & FULLER, P. M. 2016. The anatomical, cellular and synaptic basis of motor atonia during rapid eye movement sleep. *J Physiol,* 594**,** 5391-414.

BERRY, R. B., BUDHIRAJA, R., GOTTLIEB, D. J., GOZAL, D., IBER, C., KAPUR, V. K., MARCUS, C. L., MEHRA, R., PARTHASARATHY, S., QUAN, S. F., REDLINE, S., STROHL, K. P., DAVIDSON WARD, S. L., TANGREDI, M. M. & AMERICAN ACADEMY OF SLEEP, M. 2012. Rules for scoring respiratory events in sleep: update of the 2007 AASM Manual for the Scoring of Sleep and Associated Events. Deliberations of the Sleep Apnea Definitions Task Force of the American Academy of Sleep Medicine. *J Clin Sleep Med,* 8**,** 597-619.

CESARI, M., HEIDBREDER, A., BERGMANN, M., HOLZKNECHT, E., HÖGL, B. & STEFANI, A. 2021. Flexor digitorum superficialis muscular activity is more reliable than mentalis muscular activity for rapid eye movement sleep without atonia quantification: A study of interrater reliability for artifact correction in the context of semiautomated scoring of rapid eye movement sleep without atonia. *Sleep,* 44.

DATE, S., SUMI, Y., FUJIWARA, K., IMAI, M., OGAWA, K. & KADOTANI, H. 2024. Polysomnographic features prior to dream enactment behaviors in isolated rapid eye movement sleep behavior disorder. *Clin Neurophysiol,* 166**,** 74-86.

DAUVILLIERS, Y., SCHENCK, C. H., POSTUMA, R. B., IRANZO, A., LUPPI, P. H., PLAZZI, G., MONTPLAISIR, J. & BOEVE, B. 2018. REM sleep behaviour disorder. *Nat Rev Dis Primers,* 4**,** 19.

DE NATALE, E. R., WILSON, H. & POLITIS, M. 2022. Predictors of RBD progression and conversion to synucleinopathies. *Curr Neurol Neurosci Rep,* 22**,** 93-104.

DODET, P., HOUOT, M., LEU-SEMENESCU, S., GAURAV, R., MANGONE, G., CORVOL, J. C., LEHÉRICY, S., VIDAILHET, M., ROZE, E. & ARNULF, I. 2024. Isolated REM sleep without atonia is not equivalent to REM sleep behavior disorder in early-stage Parkinson's disease. *Mov Disord,* 39**,** 1190-1202.

DRAKATOS, P., DUNCAN, I., WASSERMAN, D., GNONI, V., O'REGAN, D., VARGA, A. W., TAN, H., ASHKAN, K., GRINBERG, L. T., PARRINO, L., FERINI-STRAMBI, L., GOADSBY, P. J., CHAUDHURI, K. R. & ROSENZWEIG, I. 2025. Differential motor signatures in isolated and narcolepsy-related REM sleep behaviour disorder: A preliminary study. *Front Neurol,* 16**,** 1749306.

FERRI, R., MANCONI, M., PLAZZI, G., BRUNI, O., VANDI, S., MONTAGNA, P., FERINI-STRAMBI, L. & ZUCCONI, M. 2008. A quantitative statistical analysis of the submentalis muscle EMG amplitude during sleep in normal controls and patients with REM sleep behavior disorder. *J Sleep Res,* 17**,** 89-100.

FERRI, R., MARELLI, S., COSENTINO, F. I., RUNDO, F., FERINI-STRAMBI, L. & ZUCCONI, M. 2013. Night-to-night variability of automatic quantitative parameters of the chin EMG amplitude (Atonia Index) in REM sleep behavior disorder. *J Clin Sleep Med,* 9**,** 253-8.

FERRI, R., RUNDO, F., MANCONI, M., PLAZZI, G., BRUNI, O., OLDANI, A., FERINI-STRAMBI, L. & ZUCCONI, M. 2010. Improved computation of the atonia index in normal controls and patients with REM sleep behavior disorder. *Sleep Med,* 11**,** 947-9.

FIGORILLI, M., FERRI, R., ZIBETTI, M., BEUDIN, P., PULIGHEDDU, M., LOPIANO, L., CICOLIN, A., DURIF, F., MARQUES, A. & FANTINI, M. L. 2017. Comparison between automatic and visual scorings of REM sleep without atonia for the diagnosis of REM sleep behavior disorder in Parkinson disease. *Sleep,* 40**,** zsw060.

FIGORILLI, M., MARQUES, A. R., VIDAL, T., DELABY, L., MELONI, M., PEREIRA, B., LAMBERT, C., PULIGHEDDU, M., DURIF, F. & FANTINI, M. L. 2020. Does REM sleep behavior disorder change in the progression of Parkinson's disease? *Sleep Med,* 68**,** 190-198.

FRAUSCHER, B., GSCHLIESSER, V., BRANDAUER, E., ULMER, H., POEWE, W. & HÖGL, B. 2009. The relation between abnormal behaviors and REM sleep microstructure in patients with REM sleep behavior disorder. *Sleep Med,* 10**,** 174-81.

FRAUSCHER, B., IRANZO, A., GAIG, C., GSCHLIESSER, V., GUAITA, M., RAFFELSEDER, V., EHRMANN, L., SOLA, N., SALAMERO, M., TOLOSA, E., POEWE, W., SANTAMARIA, J. & HÖGL, B. 2012. Normative EMG values during REM sleep for the diagnosis of REM sleep behavior disorder. *Sleep,* 35**,** 835-47.

GAGNON, J. F., PETIT, D., FANTINI, M. L., ROMPRÉ, S., GAUTHIER, S., PANISSET, M., ROBILLARD, A. & MONTPLAISIR, J. 2006. REM sleep behavior disorder and REM sleep without atonia in probable Alzheimer disease. *Sleep*, 29**,** 1321-5.

HÖGL, B., STEFANI, A. & VIDENOVIC, A. 2018. Idiopathic REM sleep behaviour disorder and neurodegeneration - an update. *Nat Rev Neurol*, 14**,** 40-55.

HUANG, J., ZHANG, J., LAM, S. P., LI, S. X., HO, C. K., LAM, V., YU, M. W. & WING, Y. K. 2011. Amelioration of obstructive sleep apnea in REM sleep behavior disorder: implications for the neuromuscular control of OSA. *Sleep,* 34**,** 909-15.

IRANZO, A., FERNÁNDEZ-ARCOS, A., TOLOSA, E., SERRADELL, M., MOLINUEVO, J. L., VALLDEORIOLA, F., GELPI, E., VILASECA, I., SÁNCHEZ-VALLE, R., LLADÓ, A., GAIG, C. & SANTAMARÍA, J. 2014. Neurodegenerative disorder risk in idiopathic REM sleep behavior disorder: study in 174 patients. *PLoS One,* 9**,** e89741.

IRANZO, A., FRAUSCHER, B., SANTOS, H., GSCHLIESSER, V., RATTI, L., FALKENSTETTER, T., STÜRNER, C., SALAMERO, M., TOLOSA, E., POEWE, W., SANTAMARIA, J. & HÖGL, B. 2011. Usefulness of the SINBAR electromyographic montage to detect the motor and vocal manifestations occurring in REM sleep behavior disorder. *Sleep Med,* 12**,** 284-8.

IRANZO, A., RATTI, P. L., CASANOVA-MOLLA, J., SERRADELL, M., VILASECA, I. & SANTAMARIA, J. 2009. Excessive muscle activity increases over time in idiopathic REM sleep behavior disorder. *Sleep,* 32**,** 1149-53.

IRANZO, A., SANTAMARIA, J. & TOLOSA, E. 2016. Idiopathic rapid eye movement sleep behaviour disorder: diagnosis, management, and the need for neuroprotective interventions. *Lancet Neurol,* 15**,** 405-19.

KUNZ, D. & MAHLBERG, R. 2010. A two-part, double-blind, placebo-controlled trial of exogenous melatonin in REM sleep behaviour disorder. *J Sleep Res,* 19**,** 591-6.

MANNI, R., TERZAGHI, M. & GLORIOSO, M. 2009. Motor-behavioral episodes in REM sleep behavior disorder and phasic events during REM sleep. *Sleep,* 32**,** 241-5.

MCCARTER, S. J., SANDNESS, D. J., MCCARTER, A. R., FEEMSTER, J. C., TEIGEN, L. N., TIMM, P. C., BOEVE, B. F., SILBER, M. H. & ST LOUIS, E. K. 2019. REM sleep muscle activity in idiopathic REM sleep behavior disorder predicts phenoconversion. *Neurology,* 93**,** e1171-e1179.

MCCARTER, S. J., ST LOUIS, E. K., SANDNESS, D. J., ARNDT, K., ERICKSON, M., TABATABAI, G., BOEVE, B. F. & SILBER, M. H. 2015. Antidepressants Increase REM Sleep Muscle Tone in Patients with and without REM Sleep Behavior Disorder. *Sleep,* 38**,** 907-17.

MIGLIS, M. G., ADLER, C. H., ANTELMI, E., ARNALDI, D., BALDELLI, L., BOEVE, B. F., CESARI, M., DALL'ANTONIA, I., DIEDERICH, N. J., DOPPLER, K., DUŠEK, P., FERRI, R., GAGNON, J. F., GAN-OR, Z., HERMANN, W., HÖGL, B., HU, M. T., IRANZO, A., JANZEN, A., KUZKINA, A., LEE, J. Y., LEENDERS, K. L., LEWIS, S. J. G., LIGUORI, C., LIU, J., LO, C., EHGOETZ MARTENS, K. A., NEPOZITEK, J., PLAZZI, G., PROVINI, F., PULIGHEDDU, M., ROLINSKI, M., RUSZ, J., STEFANI, A., SUMMERS, R. L. S., YOO, D., ZITSER, J. & OERTEL, W. H. 2021. Biomarkers of conversion to α-synucleinopathy in isolated rapid-eye-movement sleep behaviour disorder. *Lancet Neurol,* 20**,** 671-684.

PULIGHEDDU, M., FIGORILLI, M., CONGIU, P., LECCA, R., CASAGLIA, E., TAMBURRINO, L., ORRÙ, R., MELONI, F. & FERRI, R. 2023. Quantification of REM sleep without atonia: A review of study methods and meta-analysis of their performance for the diagnosis of RBD. *Sleep Med Rev,* 68**,** 101745.

ROGUSKI, A., RAYMENT, D., WHONE, A. L., JONES, M. W. & ROLINSKI, M. 2020. A Neurologist's Guide to REM Sleep Behavior Disorder. *Front Neurol,* 11**,** 610.

RÖTHENBACHER, A., CESARI, M., DOPPLER, C. E. J., OKKELS, N., WILLEMSEN, N., SEMBOWSKI, N., SEGER, A., LINDNER, M., BRUNE, C., STEFANI, A., HÖGL, B., BIALONSKI, S., BORGHAMMER, P., FINK, G. R., SCHOBER, M. & SOMMERAUER, M. 2022. RBDtector: an open-source software to detect REM sleep without atonia according to visual scoring criteria. *Sci Rep,* 12**,** 20886.

SINGH, A., WILLIAMS, S., CALABRESE, A. & RIHA, R. 2023. Tonic REM sleep muscle activity is the strongest predictor of phenoconversion risk to neurodegenerative disease in isolated REM sleep behaviour disorder. *J Sleep Res,* 32**,** e13792.

TAKEUCHI, N., UCHIMURA, N., HASHIZUME, Y., MUKAI, M., ETOH, Y., YAMAMOTO, K., KOTORII, T., OHSHIMA, H., OHSHIMA, M. & MAEDA, H. 2001. Melatonin therapy for REM sleep behavior disorder. *Psychiatry Clin Neurosci,* 55**,** 267-9.

WINKELMAN, J. W. & JAMES, L. 2004. Serotonergic antidepressants are associated with REM sleep without atonia. *Sleep,* 27**,** 317-21.
